# Pathogenicity of *P. gingivalis* strains carrying clusters of specific polymorphic variants of peptidylarginine deiminase gene

**DOI:** 10.3389/fcimb.2026.1820133

**Published:** 2026-05-12

**Authors:** Karolina Strzelec, Agata Dziedzic-Kowalska, Klara Ferenc, Katarzyna Łazarz-Bartyzel, Iwona Olszewska-Czyż, Iwona Rąpalska, Łukasz Sieroń, Małgorzata Aptekorz, Małgorzata Romanik, Barbara Mendrek, Natalia Oleszko-Torbus, Agnieszka Kowalczuk, Damian Kusz, Tomasz Kaczmarzyk, Marta Cześnikiewicz-Guzik, Paweł Niemiec, Tomasz J. Wasik, Katarzyna Gawron

**Affiliations:** 1 Department of Medical Microbiology, Faculty of Medical Sciences in Katowice, Medical University of Silesia, Katowice, Poland; 2 Department of Molecular Biology, Faculty of Medical Sciences in Katowice, Medical University of Silesia, Katowice, Poland; 3 Department of Periodontology, Preventive Dentistry and Oral Pathology, Faculty of Medicine, Medical College, Jagiellonian University, Krakow, Poland; 4 Department of Oral Surgery, Faculty of Medicine, Medical College, Jagiellonian University, Krakow, Poland; 5 Centre of Polymer and Carbon Materials, Polish Academy of Sciences, Zabrze, Poland; 6 Department of Orthopedics and Traumatology, Faculty of Medical Sciences in Katowice, Medical University of Silesia, Katowice, Poland; 7 Department of Biochemistry and Medical Genetics, Faculty of Health Sciences in Katowice, Medical University of Silesia, Katowice, Poland

**Keywords:** cluster, *P. gingivalis*, pathogenicity, peptidylarginine deiminase, periodontitis, polymorphic variants, virulence factors

## Abstract

**Introduction:**

Periodontitis (PD) is one of the most prevalent human inflammatory diseases of bacterial etiology, affecting over 50% of adult population worldwide. It is initiated by bacterial dysbiosis and bacteria-elicited inflammation. Among key periopathogens, the anaerobic bacterium, *P. gingivalis* has attracted a special interest. It secretes peptidylarginine deiminase (PPAD), an enzymatic protein, which due to the conversion of arginine to citrulline residue may alter protein structure and antigenicity. Despite increasing evidence supporting involvement of *PPAD* in pathogenesis of PD, functional and clinical significance of ppad gene variability remains poorly understood. The goal of this work was analysis of genetic variability of ppad gene from recently collected PD cohort in the context of *P. gingivalis* virulence and host-pathogen interactions.

**Methods:**

Clinical isolates from gingival crevicular fluid (GCF) of 35 PD patients and 20 healthy donors were analyzed for the presence, sequence variability, expression, and enzymatic activity of the ppad gene in *P. gingivalis* using PCR, BLAST, qRT-PCR, and colorimetric assays. Virulence was assessed in murine osteoblasts (MC3T3-E1) and primary human gingival fibroblasts (PHGFs) infected with *P. gingivalis* clinical strains, followed by analysis of proinflammatory cytokines, PGE2-dependent pathway enzymes, and the bone resorption marker OPG.

**Results:**

The *ppad* gene of *P. gingivalis* strains revealed pronounced variability in PD patients, affecting gene expression and enzymatic activity. Three predominant clusters of specific polymorphic variants were identified: i) S191F + N291D, ii) S191F + N291D + S528G, iii) S203P + G231N, E232T, N235D + N291D + A515V + S528G. Infection with strains harboring the S203P + G231N, E232T, N235D + N291D + A515V + S528G resulted in elevated PPAD activity, which correlated with clinical indicators of disease severity, induced COX-2 (~6-fold) and *IL-1b* (~7-fold) expression in osteoblasts and led to 94% reduction in OPG. Similarly, these strains triggered upregulation of *TNF-α*, *IL-6*, *COX-1*, and *COX-2* in PHGFs compared to the *P. gingivalis* ATCC 33277.

**Discussion:**

Collectively, these findings underscore that clusters of specific polymorphic variants of ppad are critical determinants of enzymatic function, host immune modulation and periodontal tissue destruction in PD.

## 1 Introduction

Periodontitis (PD) is one of the most prevalent human inflammatory diseases, affecting over 50% of the adult population worldwide. It is manifested by gingival bleeding, oedema, periodontal ligament degradation, attachment loss and alveolar bone resorption leading to formation of periodontal pockets and tooth exfoliation, if left untreated (Tonetti et al., 2017).

Considering the heterogeneity of the clinical course and fluctuation of disease progression, the concept on PD etiology assumes that it involves multiple causal components, which interact with each other simultaneously (Heaton and Dietrich, 2012). In general, environmental factors, such as many years of subgingival bacterial biofilm accumulation and unfavorable life style behaviors (*e.g.*, smoking, poor diet, none or irregular dental visits) in majority contribute to PD in adult and older patients. In opposite, PD in the younger population is to a greater extent dependent on genetic factors (Bergström, 2003; Heaton and Dietrich, 2012; Socransky et al., 1998; Sokransky and Haffajee, 2005; Van der Velden et al., 2011).

Although PD involves a complex pathogenesis, it is commonly thought that erosion of the soft and hard tissues supporting teeth is initiated by the spreading of subgingival biofilm, enhanced colonization of periodontal gingival pockets by bacterial species, comprising members of the “red complex”, and bacteria**-**elicited inflammation (Signat et al., 2011; Socransky et al., 1998). Among key periopathogens, the anaerobic bacterium, *Porphyromonas gingivalis* has attracted interest due to its virulence and effective strategies of immune evasion (Ferenc et al., 2026; Hajishengallis, 2014).

The bacterium expresses multiple virulence factors, including lipopolysaccharides (LPS), gingipains, fimbriae, and peptidylarginine deiminase (PPAD), an enzyme unique to *Porphyromonas* species, which catalyzes the conversion of arginine residues to citrulline, which alters protein structure and antigenicity. PPAD has been implicated in the modulation of neutrophil responses, disruption of epithelial barriers, degradation of extracellular matrix proteins, and inhibition of anaphylatoxin C5a activity (Bielecka et al., 2014; Bostanci et al., 2013; Gully et al., 2014). These activities may contribute to tissue destruction and impaired immune defense in the periodontium. It has also been shown that PPAD activity affects *P. gingivalis* adhesion to and invasion of gingival fibroblasts and promotes activation of prostaglandin E_2_ (PGE_2_)**-**dependent pathway in infected gingival fibroblasts (Gawron et al., 2014). In addition, citrullination of epidermal growth factor (EGF) by PPAD inhibits its activity and may impair tissue healing and regeneration processes during PD (Pyrc et al., 2013).

The genetic diversity of *P. gingivalis* strains, particularly polymorphic variants of the *ppad* gene, has recently gained attention. Bereta et al. (2024) identified a hyperactive PPAD variant containing 7**-**nucleotide substitutions and inducing three amino acids substitution (G231N, E232T, N235D) in protein sequence, which exhibited approximately double the enzymatic activity of the reference strain ATCC 33277 and was detected in about 25% of *P. gingivalis* strains from advanced PD (Bereta et al., 2024). Moreover, this polymorphic variant significantly induced the production of proinflammatory cytokines by gingival fibroblasts (Strzelec et al., 2025). In this study, we identified clusters of specific polymorphic variants of the *ppad* gene expressed by *P. gingivalis* strains from PD patients, and demonstrated their association with elevated expression and enhanced enzymatic activity of PPAD, worse clinical condition of periodontium and amplified host inflammatory and bone**-**related responses in PD. These results highlight the importance of genetic variability of PPAD in *P. gingivalis* pathogenicity and may at least in part explain differences in clinical outcomes of PD.

## 2 Materials and methods

### 2.1 Periodontal examination of study subjects and sample collection

This work was carried out in accordance with the Declaration of Helsinki and was approved by the Bioethics Committee of the Jagiellonian University, Medical College in Krakow, Poland (KBET/310/B/2012 and 1072.6120.156.2019). PD and control donors recruitment in the study, clinical examination and sample collection were carried out at the Department of Periodontology, Preventive Dentistry and Oral Pathology, and the Department of Oral Surgery, Medical College, Jagiellonian University, Krakow, Poland. All participants received information on the inclusion and exclusion criteria, as previously described (Gawron et al., 2019; Strzelec et al., 2025), read and signed a written informed consent form prior to inclusion in the study. The study group consisted of 35 patients diagnosed with PD (aged 32**-**80), and the control group comprising 20 healthy donors (aged 18**-**37), with no signs of oral inflammation or systemic diseases, qualified for orthodontic treatment. Medical and dental histories of all donors enrolled in the study were reviewed by periodontologists. PD diagnosis was confirmed by oral radiographs and periodontal examination of approximal plaque index (API), bleeding on probing (BOP), probing pocket depth (PPD) and clinical attachment loss (CAL). Study participants were instructed to abstain from eating and drinking from 11:00 p.m. of the night before sample collection, and to brush their teeth in the evening before the visit, but not in the morning of sampling. Gingival crevicular fluid (GCF) was collected from five inflamed periodontal pockets per each PD patient and from five random gingival crevices from each control donor. All samples collected per each study donor were pooled and used to grow of one representative *P. gingivalis* strain, as described elsewhere (Bereta et al., 2024; Strzelec et al., 2025). Gingival biopsies (1 × 1 mm per donor) were collected from representative PD and control donors in cold Dulbecco’s Modified Eagle Medium (DMEM, Gibco, Waltham, MA, USA) with antibiotics and nystatin (Sigma**-**Aldrich, Poland) for prompt cells isolation.

### 2.2 *P. gingivalis* growth from GCF

GCF samples from PD and control donors were diluted with sterile phosphate-buffered saline (PBS) (1:2) and cultured for 10**-**14 days on agar plates with brain heart infusion (BHI), supplemented with 5% defibrinated sheep blood, 5 g/L yeast extract, 0.5 mg/mL L-cysteine, 10 μg/mL hemin, and 0.5 μg/mL menadione in anaerobic conditions (85% N_2_, 10% CO_2_, 5% H_2_). Black-pigmented colonies were then subcultured into fresh blood agar plates, with passages repeated every 7**-**10 days until homogeneous cultures were obtained (Bereta et al., 2024; Gawron et al., 2014; Strzelec et al., 2025), then preserved in BHI with 20% glycerol and stored at **-**80 °C for further analyses.

### 2.3 Isolation of *P. gingivalis* genomic DNA

Genomic DNA was extracted from black pigment producing bacterial colonies, presumptively *P. gingivalis* strains, using the GeneJET Genomic DNA Purification Kit (Thermo Fisher Scientific, Waltham, MA, USA) according to the manufacturer’s instructions. Briefly, bacteria were collected from BHI agar plates using a sterile loop and suspended in PBS to obtain approximately 2 × 10^9^ cells, as estimated by optical density measurement at 600 nm (OD_600_). Samples were centrifuged at 5,000 rcf for 10 min, supernatant was discarded, and obtained pellet was resuspended in 180 μL of digestion solution with 20 μL proteinase K. The suspension was mixed and incubated at 56 °C for 30 min in a shaking thermoblock to ensure complete lysis. Next, 20 μL of RNAse A solution was added, mixed, and incubated for 10 min at RT, and then 200 μL of lysis solution was added, mixed for 15 s, then 400 μL of 50% ethanol was added and mixed again. The lysates were transferred to purification columns (GeneJET kit) and centrifuged at 6,000 rcf for 1 min. The flow**-**through was discarded, and the column was transferred to a new collection tube. A total of 500 μL of wash buffer (with ethanol) was added and centrifuged at 8,000 rcf for 1 min. After removing the filtrate, a second wash step with 500 μL of wash buffer was performed, followed by centrifugation at ≥12,000 rcf for 3 min. If any buffer remained in the column, an additional 1 min centrifugation was applied. Next, 200 μL of elution buffer was added directly to the membrane, incubated for 2 min at RT, and centrifuged at 8,000 rcf for 1 min. Purified DNA was stored at **-**20 °C until further analysis.

### 2.4 Confirmation of *P. gingivalis* species

Genomic DNA from black pigment producing bacterial strains, initially indicating *P. gingivalis* species was used for PCR with specific primers targeting the *16S rRNA* and *ppad* genes (primer sequences listed in **Supplementary Table 1**, **Supplementary Data**). PCR was performed using 4 μL of bacterial genomic DNA, 1 μM of each primer and DreamTaq Green PCR Master Mix (Thermo Fisher Scientific, Waltham, MA, USA) in a total of 12 μL reaction volume. Reaction profiles used were as follows: i) 3 min at 95 °C, ii) 40 cycles of 30 s at 95 °C, 30 s at 60 °C, 60 s (*16S rRNA*)/2 min (*ppad*) at 72 °C, and iii) final extension for 5 min at 72 °C (Bereta et al., 2024; Strzelec et al., 2025). *P. gingivalis* species was identified by electrophoretic separation of PCR products in 1.2% agarose gel stained with ethidium bromide in TAE buffer (45 min, 90 V). Amplicons corresponding to *16S rRNA* and *ppad* genes were visualized under UV light and documented using a SYNGENE gel documentation system (Cambridge, UK).

### 2.5 Sequencing and *ppad* gene sequences analysis

Samples for sequencing of the *ppad* gene of *P. gingivalis* were prepared using the GeneJET Gel Extraction Kit (Thermo Fisher Scientific, Waltham, MA, USA). Bacterial genomic DNA was amplified using Phusion High-Fidelity DNA Polymerase (Thermo Fisher Scientific, Waltham, MA, USA). PCR reaction was performed in a total volume of 50 μL containing HF buffer supplied with Phusion polymerase, 200 ng of genomic DNA as a template, and appropriate primers (**Supplementary Table 1**, **Supplementary Data**) under following conditions: i) 30 s at 98 °C, ii) 40 cycles of 10 s at 98 °C, 15 s at 61 °C, 1 min at 72 °C, iii) 5 min at 72 °C. Following amplification, 10 μL of loading buffer was added to each reaction mixture, and PCR products were separated by electrophoresis on 1% agarose gels containing ethidium bromide in TAE buffer for 45 min at 90 V. The DNA bands were visualized under UV light to identify fragments corresponding to the expected size of the amplified *ppad* gene, the appropriate bands were excised from the gel using a sterile scalpel and transferred to microcentrifuge tubes.

DNA purification was performed using the GeneJET Gel Extraction Kit (Thermo Fisher Scientific, Waltham, MA, USA). Briefly, the excised gel fragments were weighed and mixed with binding buffer (1:1). Samples were incubated for 10**-**15 min at 50**-**60 °C in a thermoblock with shaking until complete gel dissolution. The obtained solution was applied to the extraction column and centrifuged at 13,000 rcf for 1 min to bind DNA to the membrane. The column was then washed with 700 μL of wash buffer and centrifuged for 1 min, followed by an additional centrifugation step to remove residual wash buffer. Purified DNA was eluted by adding 50 μL of elution buffer, incubation for 2**-**3 min at RT, and centrifugation for 1 min at 13,000 rcf. Concentration of DNA samples was determined using a NanoDrop spectrophotometer (Thermo Fisher Scientific, Waltham, MA, USA). Control agarose gel electrophoresis was performed to assess the quality of purified products. Purified *ppad* amplicons were sent to Genomed S.A. (Warsaw, Poland) for sequencing using *ppad*-specific primers (*ppad*_FOR and *ppad*_REV; **Supplementary Table 1**, **Supplementary Data**). Accession numbers of sequences obtained by this study are listed in **Supplementary Table 2** (**Supplementary Data**).

### 2.6 Analysis of *ppad* mRNA expression

Total RNA from *P. gingivalis* strains was obtained using TRIzol reagent (Invitrogen, Waltham, MA, USA) according to the manufacturer’s protocol. Briefly, 0.5 mL of bacterial cells lysate was mixed with 100 μL of chloroform, vortexed, incubated at RT for 5 min, and centrifuged at 12,000 rcf for 15 min at 4 °C. The aqueous phase was mixed with 250 μL of isopropanol, incubated for 10 min at RT, and frozen at **-**20 °C for 2 h. After thawing and centrifugation (12,000 rcf, 15 min, 4 °C), the RNA pellet was washed twice with ice**-**cold 70% ethanol, centrifuged at 12,000 rcf for 10 min at 4 °C, air-dried, and resuspended in 20 μL of nuclease**-**free water. To remove residual DNA, samples were incubated for 30 min at 37 °C with Turbo DNAse (Invitrogen, Waltham, MA, USA), and RNA was re**-**extracted with TRIzol, as described above. Concentration and purity of obtained bacterial RNA samples were assessed by NanoDrop spectrophotometer (Thermo Fisher Scientific, Waltham, MA, USA) and reverse transcribed into cDNA. For cDNA synthesis, 400 ng of total bacterial RNA was diluted in nuclease**-**free water to a final volume of 10 μL, mixed with 2 μL of random hexamers (10×) and incubated at 70 °C for 10 min, and then cooled on ice. The reverse transcription mix (High-Capacity cDNA Reverse Transcription Kit, Invitrogen, Waltham, MA, USA) containing 1 μL of reverse transcriptase, 2 μL of 10× reverse transcription buffer, 0.8 μL of 10 mM dNTP mix and 4.2 μL of nuclease**-**free water were added to samples, and reaction was carried out at 37 °C for 2 h, followed by inactivation at 85 °C for 5 min. The resulting cDNA samples were stored at **-**20 °C.

Quantitative Real-Time PCR (qRT-PCR) was performed using 2 μL of cDNA, 0.5 μM of each primer specific for *ppad* gene (**Supplementary Table 1**, **Supplementary Data**), and 6 μL of 2× SYBR Green Master Mix (Merck, Darmstadt, Germany). The amplification consisted of initial denaturation at 95 °C for 3 min, followed by 40 cycles of denaturation at 95 °C for 30 s, annealing at 60 °C for 30 s and at 72 °C for 60 s, and extension at 72 °C for 30 s. For relative quantification of *ppad* gene expression, the ΔΔCt method was used with the reference *P. gingivalis* ATCC 33277 strain as the calibrator and the expression was normalized to the constitutive expression of *16S rRNA* gene. Reactions were run on a LightCycler 480 II (Roche, Basel, Switzerland) employing the melting curve analysis between 60**-**95 °C.

### 2.7 PPAD activity assay

The enzymatic activity assay was performed as previously described (Bereta et al., 2024; Boyde and Rahmatullah, 1980). In brief, *P. gingivalis* strains were grown in supplemented, liquid BHI medium in anaerobic conditions at 37 °C for 20**-**24 h. Next, new cultures in fresh medium were started from OD_600_ = 0.1 and incubated overnight, as above. Each culture was washed in PBS and adjusted to OD_600_ = 1 in PBS. To assay activity, 10 μL of each bacterial strain suspension was mixed with 40 μL of reaction buffer (100 mM Tris**-**HCl, pH 7.5, 5 mM dithiothreitol, and 10 mM *N***-**acetyl**-**L**-**arginine as a substrate) and incubated for 1 h at 37 °C. The reaction was stopped using 10 μL of 5 M perchloric acid. Produced citrulline was measured using a colorimetric method and compared with a standard curve for L**-**citrulline. Citrulline detection reagent, consisting of 1 part of solution A (0.5% 2,3-butanedione monoxime, 0.01% thiosemicarbazide) and 2 parts of solution B (0.25 mg/mL FeCl_3_, 24.5% sulphuric acid, 17% orthophosphoric acid) was prepared immediately before use and added to each analyzed and standard curve sample, followed by incubation at 110 °C for 20 min, and absorbance measurement at 535 nm. The amount of citrullinated product was calculated using the standard curve and expressed relative to the reference *P. gingivalis* ATCC 33277 strain.

### 2.8 Pre-osteoblasts culture to osteoblasts

Mouse pre**-**osteoblasts (MC3T3**-**E1, subclone 4**-**CRL**-**2593, ATCC) were cultured in α**-**MEM (Gibco, Waltham, MA, USA) supplemented with 10% heat**-**inactivated fetal bovine serum (FBS), 50 U/mL penicillin, and 50 μg/mL streptomycin at 37 °C in a humidified atmosphere with 5% CO_2_. Cells were maintained in T75 flasks until 90**-**95% confluence, then passaged and seeded into 6**-**well plates (200,000 cells/well) and 24**-**well plates (30,000 cells/well). After ~20 h, culture medium was replaced with α**-**MEM with 10% heat**-**inactivated FBS, 50 U/mL penicillin, and 50 μg/mL streptomycin supplemented with osteogenic factors, *i.e.*, 10^-8^ M dexamethasone, 2 mM β**-**glycerophosphate, and 20 μg/mL ascorbic acid (magnesium salt). Differentiation procedure was carried out for 28 days; supernatants and cell lysate samples were collected on the day “0” (control, no differentiation factors added), 7^th^, 14^th^, 21^st^ and 28^th^ and were used to assess osteoblastic cells markers expression during differentiation. Osteoblasts at 28^th^ day of differentiation were washed with sterile PBS and maintained in α**-**MEM containing 2% FBS (without antibiotics) for infection experiments with *P. gingivalis* strains.

### 2.9 PHGFs isolation and culture

PHGFs were isolated from gingival biopsies of 2 representative PD donors and 1 control donor using enzymatic method (dispase and collagenase I, Invitrogen, Waltham, MA, USA) as reported elsewhere (Gawron et al., 2018, 2017a, 2014). Cells were cultured in DMEM (Gibco, Waltham, MA, USA) with high glucose, supplemented with 10% heat**-**inactivated FBS, 50 U/mL penicillin, and 50 μg/mL streptomycin at 37 °C in a humidified atmosphere with 5% CO_2_ until 95% confluency. Cells were washed with sterile PBS and detached from the culture vessel using 3 mL of 0.05% trypsin/ethylenediaminetetraacetic acid (EDTA) for 5 min at 37 °C. Trypsinization was neutralized with 3 mL DMEM with 10% FBS and cell suspension was centrifuged at 300 rcf for 10 min at 4 °C. Cell pellet was then resuspended in 3 mL of culture medium, diluted in trypan blue (1:10), and cell counts were determined using a TC20 Automated Cell Counter (Bio**-**Rad, San Francisco, CA, USA). For infection experiments, cells were seeded in 24**-**well plates at a density of 250,000 cells per well and cultured for ~ 20 h. Next day, prior the infection, culture medium was removed, cells were washed with sterile PBS, and replaced with antibiotic**-**free DMEM containing 2% FBS.

### 2.10 Preparation of *P. gingivalis* strains for cells infection

Bacterial colonies were transferred from agar plates into 10 mL of liquid BHI medium and incubated for approximately 20 h at 37 °C in an anaerobic chamber. The optical density (OD_600_) of the culture was then measured and adjusted to 0.1 in fresh BHI medium to a final volume of 15 mL, and incubated for ~20 h at 37 °C under anaerobic conditions. Following incubation, bacterial cells were centrifuged at 4,500 rcf for 10 min at 4 °C and washed with sterile PBS. The centrifugation and washing with PBS were repeated three times. Finally, the bacterial cells pellet was resuspended in 5 mL of PBS, the optical density at OD_600_ was measured, and bacterial suspension in PBS with OD_600_ = 1 was prepared. For infection, the reference strain ATCC 33277, clinical strains and control laboratory mutant (C351A) of *P. gingivalis* were used. The experiments were carried out at a multiplicity of infection (MOI) of 1:100 for 24 h in α**-**MEM for MC3T3**-**E1 mouse osteoblasts (28^th^ day of differentiation) or in DMEM for PHGFs, both containing 2% FBS without antibiotics (Gawron et al., 2014; Lagosz-Cwik et al., 2021; Su et al., 2024; Zhang et al., 2010). Following infection experiments, culture supernatants and cell lysates were collected and stored at **-**20 °C/**-**80 °C for further analyses.

### 2.11 Measurement of cell viability

Cell viability was assessed using the MTT (Merck, Darmstadt, Hesse, Germany). After cells infection (24 h, MOI 100) with *P. gingivalis* strains (ATCC 33277, clinical strains and control mutant C351A), 100 μL of 3-(4,5-dimethylthiazol-2-yl)-2,5-diphenyltetrazolium bromide (MTT) dye (1.5 mg/mL in PBS) was added to the cells and the plates were incubated at 37 °C for 2 h. Next, the medium was discarded and 500 μL of dimethylsulfoxide were added to each well and the plates were shaken to facilitate dissolving of formazan crystals. As controls uninfected cells were used. Absorbance values were measured at OD_560_ on a Victor X5 plate reader (PerkinElmer, Waltham, MA, USA). The MTT assay was performed in three replicates.

### 2.12 Gene expression in mammalian cells infected with *P. gingivalis*

#### 2.12.1 RNA isolation

RNA isolation was performed with the use of TRIzol reagent (Invitrogen, Waltham, MA, USA). To 0.5 mL of cell lysate, 100 μL of chloroform was added, vortexed for 10 s, incubated for 5 min at RT, and centrifuged at 12,000 rcf for 15 min at 4 °C. The aqueous phase was mixed with 250 μL of isopropanol, incubated for 10 min at RT and frozen at **-**20 °C for ~ 2 h. After thawing, samples were centrifuged at 12,000 rcf for 15 min at 4 °C, the supernatant was removed, and the RNA pellet was washed twice with 200 μL of ice-cold 70% ethanol, followed by centrifugation at 12,000 rcf for 10 min at 4 °C. Ethanol was discarded, and the RNA pellet was air**-**dried and resuspended in 20 μL of nuclease**-**free water. To eliminate residual genomic DNA, samples were digested for 30 min at 37 °C with 1 μL of Turbo DNAse mixed with 5 μL of 10× Turbo DNAse buffer and 25 μL of nuclease**-**free water (Invitrogen, Waltham, MA, USA). Subsequently, 0.5 mL of TRIzol was added to each sample, and the RNA isolation procedure was repeated, as described above. The RNA concentration and purity were assessed using a NanoDrop spectrophotometer (Thermo Fisher Scientific, Waltham, MA, USA).

#### 2.12.2 Reverse transcription reaction

Reverse transcription was performed using High**-**Capacity cDNA Reverse Transcription Kit (Invitrogen, Waltham, MA, USA). A total of 400 ng of RNA was resuspended in nuclease**-**free water to a final volume of 10 μL, followed by the addition of 2 μL of 10× random hexamers. The samples were incubated at 70 °C for 10 min, cooled to 25 °C, and placed on ice. Next, 8 μL of master mix, containing 1 μL of reverse transcriptase, 0.8 μL of 10 mM dNTP mix, 2 μL of 10× reverse transcriptase buffer and 4.2 μL of nuclease**-**free water was added to each reaction sample. The reverse transcription was carried out at 37 °C for 2 h, followed by enzyme inactivation at 85 °C for 5 min. The resulting cDNA was stored at **-**20 °C until further use.

#### 2.12.3 Quantitative real-time PCR

The qRT**-**PCR was performed using 2 μL of 5× diluted cDNA template, 6 μL of reaction mixture containing 2× SYBR Green Master Mix (Merck, Darmstadt, Germany), 1 μL of standardized primers sets (**Supplementary Table 1**, **Supplementary Data**) and 1 μL of nuclease**-**free water. The reaction profile used was as follows: i) 3 min at 95 °C, ii) 40 cycles of 30 s at 95 °C, 30 s at 57 °C, 45 s at 72 °C, iii) 30 s at 72 °C. For relative quantification of analyzed genes expression the ΔΔCt method was used. The housekeeping gene *ACTB* (β**-**actin, huACTB/moACTB) served as an internal control. Amplification of specific products was performed with a LightCycler 480 II system (Roche, Basel, Switzerland).

### 2.13 ELISA and colorimetric assays

To assess proliferation of pre**-**osteoblasts and markers of osteoblastic cells, ELISA and colorimetric assays were used. To this aim, cells at 7^th^, 14^th^, 21^st^ and 28^th^ day of culture in the presence of osteogenic factors and control cells (day “0”, w/o osteogenic factors) were used. Concentration of osteoprotegerin (OPG) was measured using the Mouse OPG/TNFRSF11B Quantikine ELISA Kit (R&D Systems, Minneapolis, MN, USA). Alkaline Phosphatase Assay Kit**-**Colorimetric (Abcam, Cambridge, UK) was used to evaluate activity of alkaline phsphatase (ALP) and calcium ions (Ca^2+^) concentration was assessed employing Calcium Colorimetric Assay Kit (Biovision, Milpitas, CA, USA). Cells proliferation was analyzed by MTT assay (Merck, Darmstadt, Hesse, Germany). All colorimetric assays were performed in accordance with the manufacturers’ protocols at the specified wavelengths using a Victor X5 plate reader (PerkinElmer, Waltham, MA, USA).

### 2.14 Microscopic analysis

For further evaluation of pre**-**osteoblasts differentiation to mature osteoblasts, formation of mineralization nodules was visualized using Alizarin Red S (Sigma-Aldrich, St. Louis, MO, USA). In brief, after washing with PBS, cells were fixed in 4% formaldehyde in PBS (pH 7.4) for 30 min at RT, and rinsed again with PBS. Staining was carried out using 0.5% Alizarin Red S solution in deionized water (pH 4.0) for 1 h at RT. Subsequently, the cells were washed three times with deionized water and dehydrated in 70% ethanol for 5**-**10 min. Images of mineralization nodules were acquired at 200× magnification and analyzed using a light microscope (Nikon Eclipse Ti, Japan) coupled with NIS-Elements F 3.0 software (Nikon Inc., Japan).

### 2.15 Statistical analysis

Data were analyzed using GraphPad Prism v. 10 (GraphPad Software, San Diego, CA, USA) and STATISTICA v. 13.0 (TIBCO Software Inc., CA, USA). The normality of the distribution of quantitative data was assessed using the Shapiro**-**Wilk test. Quantitative data are presented as means ± SD or means ± SEM. For comparisons of normally distributed variables, one**-**way analysis of variance (ANOVA) was applied, followed by Tukey’s *post-hoc* test, if significant differences were detected. For variables with non-normal distributions, the Kruskal**-**Wallis test followed by Dunn’s *post hoc* test was used. The relationships between quantitative variables were assessed using Pearson’s correlation coefficient (r), as the variables were normally distributed. The χ² test was used to compare genotype variant frequencies across categories of qualitative variables. Yates’ correction was applied for subgroups with fewer than ten subjects. Cases with missing data were excluded from the relevant analyses. The differences were considered statistically significant at *p* < 0.05.

## 3 Results

### 3.1 Clinical classification of PD

The study group included 35 patients with PD (25 women and 10 men; mean age: 60 years; age range: 32**-**80 years). Control group consisted of 20 donors with healthy periodontium (19 women and 1 man; mean age: 26 years; age range: 18**-**37 years). Women predominated in both groups, representing 71% of PD patients (25/35) and 95% of healthy donors (19/20) (**Table 1**).

**Table 1:** Demographic data of PD group stratified by stages and severity *vs.* CTRL.

PD (N = 35)				
PD classification	Number of donors	Age	Gender	
			Female	Male
Stage II Grade A	1	33	1	0
Stage II Grade B	1	70	1	0
Stage III Grade A	12	37**-**72	9	3
Stage III Grade B	7	32**-**70	6	1
Stage III Grade C	1	68	0	1
Stage IV Grade A	4	56**-**78	3	1
Stage IV Grade B	1	70	1	0
Stage IV Grade C	8	34**-**80	4	4
CTRL (N = 20)				
Healthy periodontium	20	18**-**37	19	1

PD, periodontitis; CTRL, controls.

Most of patients enrolled in the study (20/35; 57%) were diagnosed with PD at Stage III (including 12 patients at Grade A, 7 **-** at Grade B, and 1 patient at Grade C, respectively). Average values of periodontal parameters obtained in this group included API of 34.5%, BOP of 52.4%, PPD **-** 4.96 mm and CAL **-** 5.35 mm. In the second**-**largest subgroup (13/35; 37%) were enrolled patients diagnosed with PD at Stage IV, which refer to the most advanced form of PD. Four patients presented PD at Grade A, 1 **-** at Grade B, and 8 patients at Grade C; mean values of periodontal parameters in this subgroup included API **-** 51%, BOP **-** 73.03%, PPD **-** 5.85 mm, and CAL **-** 7.28 mm. The least numerous subgroup comprised patients with PD at Stage II (2/35; 6%) (1 patient at Grade A, and 1 **-** at Grade B), corresponding to moderate PD. Mean values of periodontal measurements were as follows, API **-** 20%, BOP **-** 45%, PPD **-** 4.7 mm, CAL **-** 4.0 mm. Control donors presented clinical parameters within reference range (mean API **-** 30.7%, BOP **-** 13.31%, PPD **-** 1.33 mm, CAL **-** 1.34 mm) (**Tables 1**, **2**).

**Table 2:** Clinical classification of PD (according AAP recommendations in 2018).

	API [%] mean (range)	BOP [%] mean (range)	PPD [mm] mean (range)	CAL [mm] mean (range)
PD (N = 35)				
Stage II Grade A**-**B (N = 2)	20 (15 **-** 25)	45 (30 **-** 60)	4.7 (4.0 **-** 5.4)	4.0 (4.0 **-** 4.0)
Stage III Grade A**-**C (N = 20)	34.5 (10 **-** 100)	52.4 (23 **-** 100)	4.96 (3.6 **-** 6.0)	5.35 (4.2 **-** 7.0)
Stage IV Grade A**-**C (N = 13)	51 (20 **-** 100)	73.03 (25 **-** 100)	5.85 (4.3 **-** 7.2)	7.28 (4.6 **-** 8.9)
CTRL (N = 20)				
Healthy periodontium	30.7 (7 **-** 81)	13.31 (0**-**38)	1.33 (0.9 **-** 1.7)	1.34 (0.9 **-** 1.7)
Reference range	0**-**10	0**-**10	1**-**3	1**-**2

API, approximal plaque index, 1 value/oral cavity; BOP, bleeding on probing, 1 value/oral cavity; PPD, probing pocket depth, 5 values/oral cavity; CAL, clinical attachment loss, 5 values/oral cavity; PD, periodontitis; CTRL, controls.

### 3.2 *16S rRNA* and *ppad* expression by *P. gingivalis*

Homogeneous, black**-**pigmented bacterial colonies resembling *P. gingivalis* were cultured from GCF samples of 35 PD patients and 20 healthy donors, enrolled in the study. For analyses, one representative clinical strain from each donor was used. *P. gingivalis* species was identified by PCR amplification of the *16S rRNA* and *ppad* genes and visualization in agarose gels. Amplicons of both markers were detected for all strains used, thus confirming *P. gingivalis* species (data not shown).

### 3.3 *In silico* analysis of polymorphic variants of *ppad* gene

Analysis of *ppad* gene from *P. gingivalis* strains performed in previous cohort of PD and healthy donors, as well as of 60 *ppad* sequences deposited in databases (GenBank, NCBI) showed significant diversity in comparison with the *ppad* sequence from wild**-**type reference *P. gingivalis* (ATCC 33277) strain, the correlation of the new G231N, E232T, N235D polymorphic variant occurrence with increased pathogenicity of *P. gingivalis* and advanced PD (Bereta et al., 2024; Strzelec et al., 2025). These observations emphasize the potential impact of genetic diversity, and particularly the occurrence of polymorphic variants in *ppad* on pathogenesis of PD. To further verify this thesis, studies were conducted employing *ppad* sequences from *P. gingivalis* strains cultured recently from a new cohort of PD patients. Two sequencing reads were obtained for each *P. gingivalis* strain to generate a consensus sequence, which was then aligned against the reference strain ATCC 33277. *In silico* analysis was performed using complete coding sequences of the *ppad* gene from all *P. gingivalis* strains, excluding the sequences containing insertions or deletions to ensure alignment consistency. Across all analyzed samples, 51 nucleotide substitutions were identified, including 44 single nucleotide changes and 1 seven**-**nucleotide substitution. Variants were classified according to two independent criteria: (i) the functional effect on the encoded protein sequence and (ii) the frequency of occurrence across analyzed samples (**Table 3**). Based on functional effect, variants were categorized as synonymous (no change in amino acid sequence) or missense (non-synonymous, resulting in an amino acid change in the protein sequence). Based on frequency, variants were defined as polymorphic variants, when present in more than 15% of sequences, whereas variants present in less than 15% of cases were considered as missense mutations. Importantly, these two classification systems are independent, and a given variant may be simultaneously characterized by its functional effect (synonymous or missense) and its frequency (polymorphic or rare). Interestingly, six novel polymorphisms of *ppad*, accounting for 13.33% of the analyzed substitutions, not previously reported in public databases were identified in *P. gingivalis* strains from PD donors, but not from controls. The most prevalent polymorphic variant was the N291D substitution, detected in 51.54% of *P. gingivalis* strains, followed by the S191F, present in 45.71% of analyzed samples. These two variants frequently co**-**occurred within individual sequences, suggesting a potential linkage or co**-**selection mechanism of occurrence. A notable observation was the co**-**occurrence of the S203P (20.00%) substitution with the seven**-**nucleotide substitutions, with the latter resulting in three amino acid G231N, E232T, N235D change of PPAD (17.14%). The G231N, E232T, N235D polymorphic variant was identified in six *P. gingivalis* strains together with the N291D variant. Notably, both variants are located close to the active center of PPAD, which may suggest a possible structural or functional association of these variants. In addition, we identified two other variants, *i.e.*, the A515V (20.00%) and S528G (28.57%) (**Table 4**). Importantly, none of the identified polymorphic variants was found in *ppad* sequences from *P. gingivalis* strains from control group, reinforcing their potential association with disease specific microbial adaptation or pathogenicity.

**Table 3:** PPAD variants produced by *P. gingivalis* strains cultured from PD and CTRL groups based on nucleotide sequence analysis.

Variant category of PPAD	PD (N = 35)	%	CTRL (N = 20)	%
Polymorphic variants	12	23.53	0	0.00
Missense (non-synonymous) variants	6	11.76	0	0.00
Synonymous variants	33	64.71	22	100.00

PPAD, peptidylarginine deiminase; PD, periodontitis; CTRL, controls.

**Table 4:** Polymorphic variants of the *ppad* gene produced by *P. gingivalis* strains from PD.

Polymorphic variant	Frequency [%]	Number of PD donors	Proximity to PPAD active center
S191F	45.71	16	Distal
S203P	20.00	7	Distal
G231N, E232T, N235D	17.14	6	Close
N291D	51.54	18	Close
A515V	20.00	7	Distal
S528G	28.57	10	Distal

PD, periodontitis; PPAD, peptidylarginine deiminase.

Taken together, these results underscore the prevalence and potential clinical relevance of specific polymorphic variants of the *ppad* gene, particularly the N291D and the G231N, E232T, N235D cluster due to their location in close proximity to the active center of PPAD.

### 3.4 Expression of *ppad* mRNA by *P. gingivalis* strains

**Figure 1:**
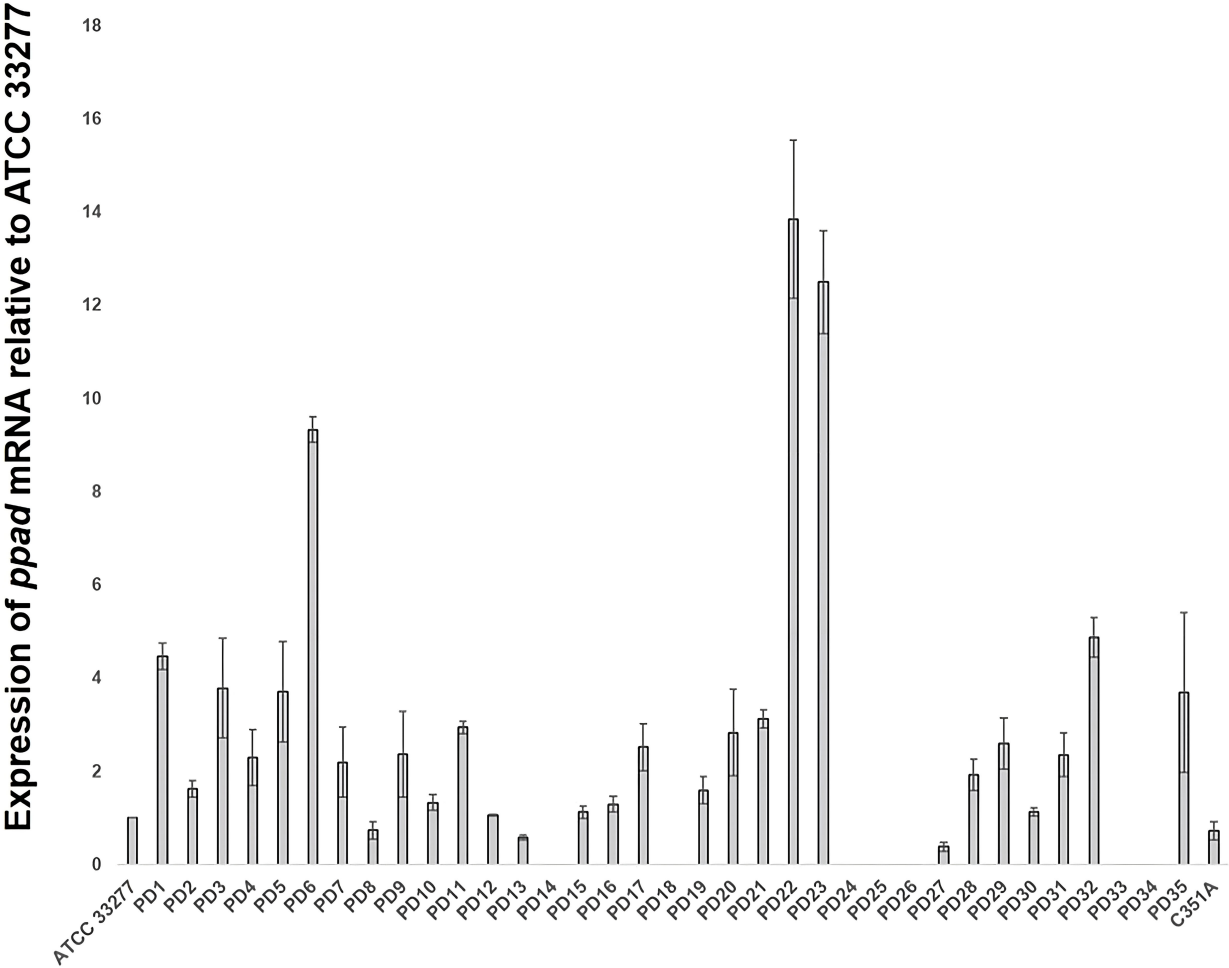
Relative mRNA expression of *ppad* in clinical *P. gingivalis* strains. Total RNA was extracted from *P. gingivalis* cells, and reverse**-**transcribed into cDNA. Expression of *ppad* mRNA is presented consecutively for clinical *P. gingivalis* strains from 35 PD donors. ATCC 33277, *P. gingivalis* reference strain (control); PD1**-**PD35, *P. gingivalis* strains obtained from 35 patients with periodontitis; C351A, a control ATCC 33277 strain, which produces a catalytically inactive form of PPAD; *ppad*, peptidylarginine deiminase gene; *16S rRNA*, a reference gene. Analysis was performed using qRT**-**PCR. Results represent the mean ± SD from three independent experiments.

As presented in **Figure 1**, more than half of *P. gingivalis* strains (24/35) exhibited an increase of relative *ppad* expression compared to the control reference strain (ATCC 33277). Among 24 strains of *P. gingivalis* producing polymorphic variants of the *ppad* gene, 18 (75.00%) demonstrated elevated expression of *ppad*. Amongst them, eleven strains cultured from PD3, PD5, PD7, PD11, PD17, PD20, PD22, PD23, PD29, PD31, and PD32 donors, respectively, showed at least a 2**-**fold increase of *ppad* expression in comparison to the reference ATCC 33277 strain. In contrast, despite the presence of viable bacteria, relative expression of *ppad* was undetectable in seven *P. gingivalis* strains collected from PD14, PD18, PD24, PD25, PD26, PD33, PD34 donors, indicating possible gene silencing or loss of expression independent of polymorphic variants. Collectively, these findings support a potential link between the presence of polymorphic variants of *ppad* gene and increased expression of PPAD.

### 3.5 PPAD activity of *P. gingivalis* strains

**Figure 2:**
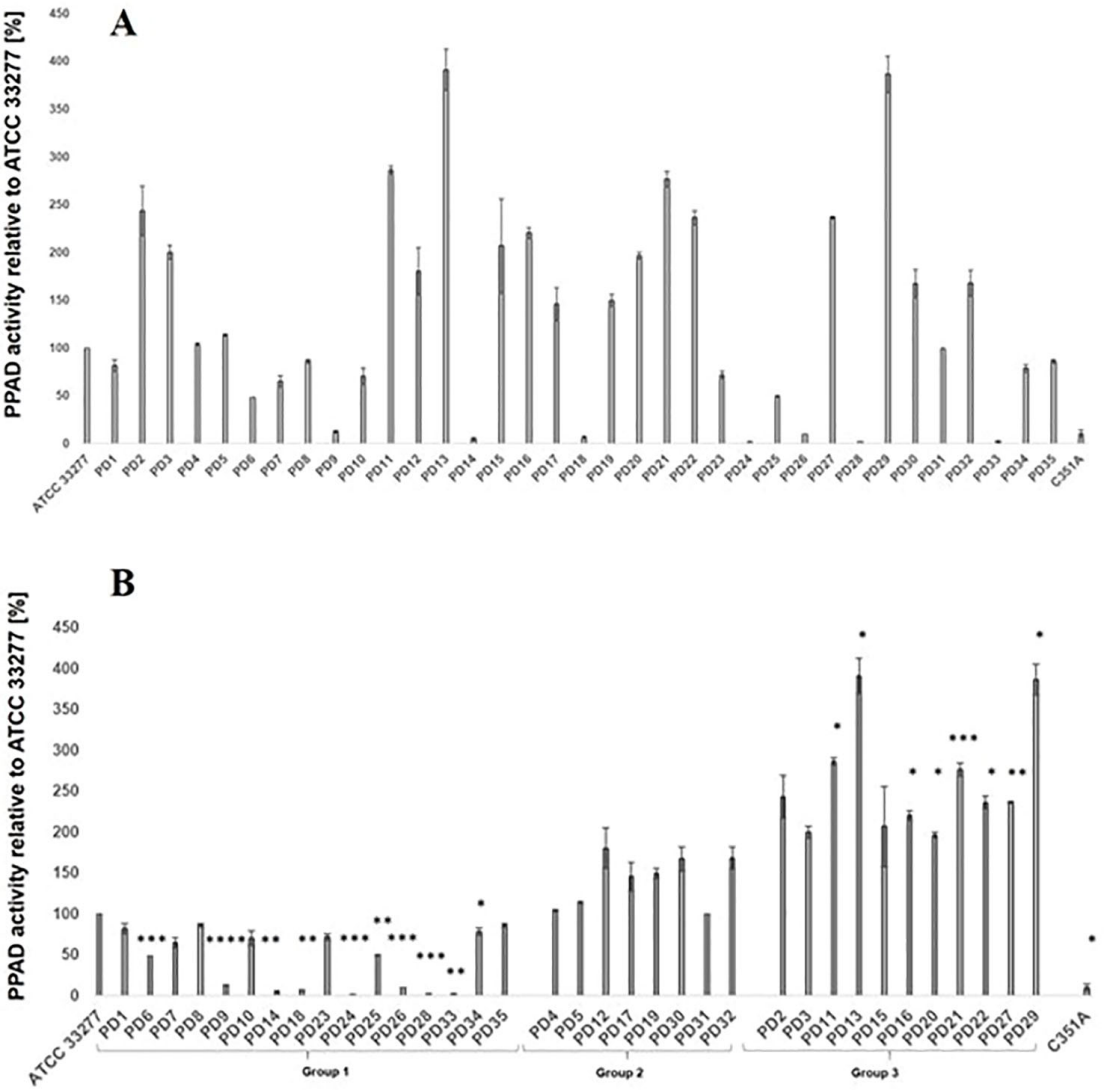
Enzymatic activity of PPAD in clinical *P. gingivalis* strains. PPAD enzymatic activity was measured using N**-**acetyl**-**L**-**arginine as a substrate in colorimetric assay, with citrulline production quantified relative to standard curve. **(A)** Enzymatic activity of PPAD presented consecutively for clinical *P. gingivalis* strains from 35 PD donors. PPAD, peptidylarginie deiminase; ATCC 33277, *P. gingivalis* reference strain with enzymatic activity set at 100% (control); PD1**-**PD35, *P. gingivalis* strains obtained from 35 patients with periodontitis; C351A, a control ATCC 33277 strain, which produces a catalytically inactive form of PPAD. Results are expressed as a percentage of activity relative to the reference ATCC 33277 strain. Data represent the mean ± SD from three independent experiments; **(B)** Enzymatic activity of PPAD in clinical *P. gingivalis* strains from 35 PD donors divided into three groups on the base of PPAD activity level, group 1: PPAD activity at 0 **-** 100%, group 2: 100 **-** 200%, group 3: **>** 200%. PPAD, peptidylarginie deiminase; ATCC 33277, *P. gingivalis* reference strain with enzymatic activity set at 100% (control); PD1**-**PD35, *P. gingivalis* strains obtained from 35 patients with periodontitis; C351A, a control ATCC 33277 strain, which produces a catalytically inactive form of PPAD; Results are expressed as a percentage of activity relative to the control ATCC 33277 strain. Data represent the mean ± SEM from three independent experiments. Statistical analysis was performed using one**-**way ANOVA followed by Tukey’s *post hoc* test in comparison to the ATCC 33277 strain. *p < 0.05; **p < 0.01; ***p < 0.001; ****p < 0.0001.

Enzymatic activity of PPAD was assessed for *P. gingivalis* strains obtained from 35 PD donors (study group) and compared with the reference *P. gingivalis* strain (ATCC 33277) as a control (**Figure 2**). The analysis revealed considerable variability in PPAD activity dependently on the type of mutation and its co**-**occurrence in the *ppad* gene. Most of *P. gingivalis* strains harboring polymorphic variants of *ppad* (16/24) derived from PD (PD2, PD3, PD11**-**13, PD15**-**17, PD19**-**22, PD27, PD29, PD30 and PD32) showed an average 1.5**-**fold increase of PPAD activity relative to the reference *P. gingivalis* strain (ATCC 33277). Unlike, clinical strains which did not harbor polymorphic variants of *ppad*, but synonymous and/or missense mutations, did not display comparable increase of PPAD activity. Moreover, seven strains of *P. gingivalis* cultured from PD9, PD14, PD18, PD24, PD26, PD28, PD33 donors revealed PPAD activity levels near the detection threshold, ranging from approximately 2% to 12% (**Figure 2A**).

To further explore the association of the occurrence of *ppad* polymorphic variants and enzymatic activity of PPAD, 35 analyzed *P. gingivalis* strains were stratified into three groups based on the range of PPAD activity, *i.e.*, group 1 (0 **-** 100%), group 2 (100% **-** 200%), and group 3 (**>** 200%) (**Table 5**, **Figure 2B**).

**Table 5:** Classification of polymorphic variants of *P. gingivalis ppad* gene from 35 PD patients on the basis of enzymatic activity of PPAD.

PD donors N [%]	PD classification	Polymorphic variants
Group 1: PPAD activity <100%		
3 [8.57]	III B IV C	S191F
2 [5.7]	III A	S191F + N291D
1 [2.86]	III A	S203P + G231N, E232T, N235D + N291D + A515V + S528G
10 [28.57]	III A III B IV C	ABSENCE
Group 2: PPAD activity 100%-200%		
1 [2.86]	II B	S191F
3 [8.57]	III B IV A IV C	S191F + N291D
1 [2.86]	III A	S191F + N291D + S528G
1 [2.86]	III A	S203P + G231N, E232T, N235D + A515V
2 [5.71]	IV A	ABSENCE
Group 3: PPAD activity > 200%		
2 [5.71]	III A III B	S191F + N291D
5 [14.28]	II A III C IV A IV C	S191F + N291D + S528G
4 [11.43]	III A III B IV B IV C	S203P + G231N, E232T, N235D + N291D + A515V + S528G

PD, periodontitis; PPAD, peptidylarginine deiminase.

The most frequently occurring in all groups of *P. gingivalis* strains were the N291D and the S191F variants (51.42%; 48.56%), then the S528G variant (31.43%), and next the S203P, A515V and the G231N, E232T, N235D (17.14% each). The S191F variant occurred as a single change in the entire *ppad* sequence of *P. gingivalis* strains classified to group 1 and 2 (11.43%), as co**-**occurring change with the N291D variant in all groups (20.00%) or co**-**occurring with the N291D and S528G in group 2 and 3 (17.14%). Enzymatic activity of *P. gingivalis* strains producing S191F variant remained below that of ATCC 33277, in case of *P. gingivalis* strains producing the S191F + N291D variants it reached 139.74%, while for *P. gingivalis* strains harboring the S191F + N291D + S528G cluster **-** 251.68%.

*P. gingivalis* strains classified into group 3 demonstrated the most elevated values of PPAD activity (**>** 200%), and 3 most frequently occurring clusters of polymorphic variants (31.42%): i) S191F + N291D (5.71%), ii) S191F + N291D + S528G (14.28%), and iii) S203P + G231N, E232T, N235D + N291D + A515V + S528G (11.43%). Notably, each of these clusters contained at least one polymorphic variant located in close proximity to the PPAD active site, most commonly N291D, but also G231N, E232T, N235D variants. *P. gingivalis* strains harboring any of these clusters showed over 2**-**fold increase of PPAD activity relative to the reference ATCC 33277. In contrast, *P. gingivalis* strains without polymorphic variants, expressing *ppad* sequence identical to the reference ATCC 33277 strain, which derived from donors PD1 and PD4 displayed activity levels close to 100%, while from PD6, PD24, PD25, and PD33 donors **-** much below that of control.

To identify *ppad* gene variants/polymorphic clusters characteristic of *P. gingivalis* strains exhibiting the highest PPAD activity (Group 3: PPAD activity > 200%), the frequencies of all *ppad* variants detected in Group 3 were compared with their frequencies in strains with enzyme activity ≤ 200% (Groups 1 and 2 combined). The clusters: S191F + N291D + S528G and S203P + G231N, E232T, N235D + N291D + A515V + S528G within the *ppad* gene, were found to occur at approximately 10-fold higher frequencies in strains with PPAD activity > 200% compared with the remaining strains (**Table 6**), and these differences were statistically significant. In contrast, the frequency of the S191F + N291D cluster was not a differentiating factor in this comparison (**Table 6**).

**Table 6:** Frequency of *ppad* gene polymorphic variants/clusters between *P. gingivalis* strains with PPAD activity > 200% and ≤ 200%.

Clusters of polymorphic variants of PPAD	PPAD activity > 200% N = 11 n (%)	PPAD activity ≤ 200% N = 24 n (%)	χ²	*p*
S191F + N291D	2 (18.18)	5 (20.83)	0.03	0.858
S191F + N291D + S528G	5 (45.46)	1 (4.17)	8.79	0.003
S203P + G231N, E232T, N235D + N291D + A515V + S528G	4 (36.36)	1 (4.17)	6.20	0.013

PPAD, peptidylarginine deiminase.

Taking together, these results demonstrated that *P. gingivalis* strains, which showed elevated values of enzymatic activity of PPAD harbor most frequently occurring clusters of specific polymorphic variants of *ppad* gene.

### 3.6 Correlation analysis of PPAD activity of *P. gingivalis* strains harboring clusters of specific polymorphic variants of *ppad* gene and clinical parameters of PD periodontium

**Figure 3:**
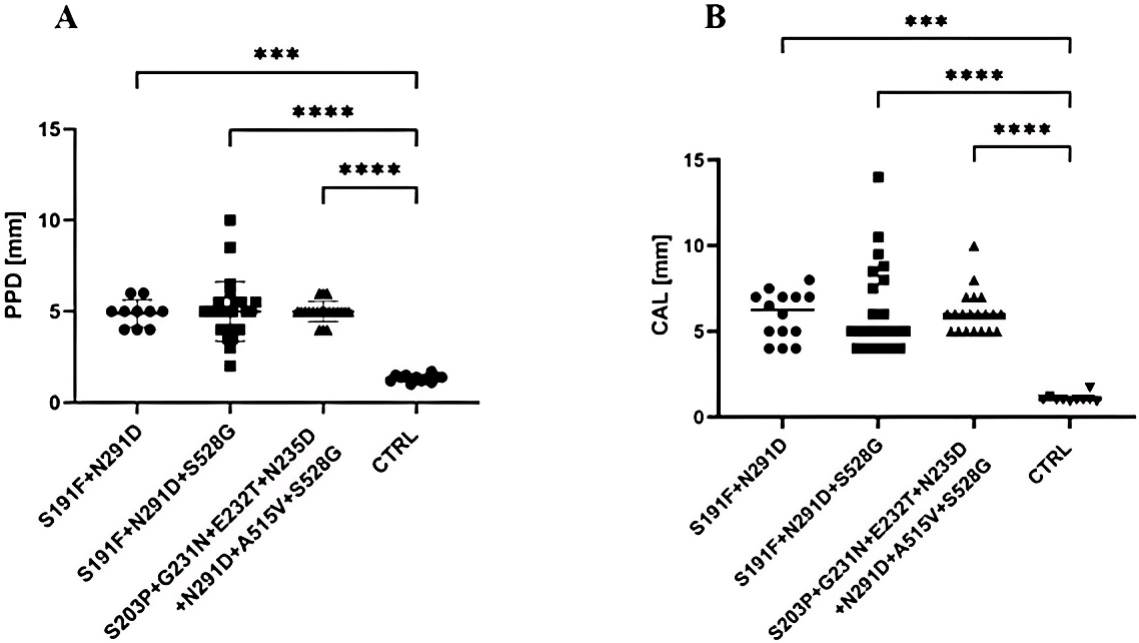
Comparative analysis of clinical parameters of periodontium from PD and control donors. Analysis included **(A)** PPD (5 values/oral cavity), and **(B)** CAL (5 values/oral cavity) of periodontium from PD patients infected with *P. gingivalis* strains harboring any of 3 most frequently occurring clusters of polymorphic variants of the *ppad* gene: i) S191F + N291D, ii) S191F + N291D + S528G, or iii) S203P + G231N, E232T, N235D + N291D + A515V + S528G and compared with parameters examined in control group (CTRL, donors with healthy periodontium), harboring *P. gingivalis* strains w/o polymorphic variants of the *ppad* gene. PPD, probing pocket depth; CAL clinical attachment loss. Data are presented as mean ± SEM. Statistical analysis was performed using the Kruskal**-**Wallis test with Dunn’s *post hoc* test. ***p < 0.001; ****p < 0.0001.

Next, the correlation analysis was performed to investigate the potential association of PPAD enzymatic activity of *P. gingivalis* strains with most frequently occurring clusters of specific polymorphic variants of *ppad* gene (group 3) (**Table 5**, **Figure 2B**) and clinical indicators of periodontal inflammation, specifically BOP, CAL, and PPD of PD patients - donors of these strains. As presented in **Table 7**, PPAD activity correlated with PPD (r = 0.61) and CAL (r = 0.81) (*p* < 0.05). Furthermore, we analyzed whether the periodontal parameters (PPD, CAL) examined for PD patients **-** donors of *P. gingivalis* strains carrying most frequently occurring clusters of specific polymorphic variants of *ppad* and presenting elevated activity of PPAD (group 3) (**Table 5**, **Figure 2B**) vary from PPD and CAL values measured for healthy donors (control group). The analyses revealed that both analyzed parameters from PD patients infected with *P. gingivalis* strains harboring any cluster of co**-**occurring variants: i) S191F + N291D, ii) S191F + N291D + S528G, or iii) S203P + G231N, E232T, N235D + N291D + A515V + S528G were significantly elevated as compared to those observed for controls, *i.e.*, donors of *P. gingivalis* strains producing identical *ppad* (sequence) to the control *P. gingivalis* (ATCC 33277) strain (**Figures 3A, B**).

**Table 7:** Correlation of PPAD activity of *P. gingivalis* strains and clinical parameters of periodontium from PD.

Correlates	PPAD activity	BOP	CAL	PPD
PPAD activity	–	0.48	0.81 *	0.61 *
BOP	0.48	–	0.43	0.78 *
CAL	0.81 *	0.43	–	0.62 *
PPD	0.61 *	0.78 *	0.62 *	–

Values of r = 1 **-** 0.7, a strong positive correlation between the two analyzed variables; r = 0.69 **-** 0.3, a moderate positive correlation; r **≤** 0.29, a weak positive correlation; r = 0, no correlation. BOP, bleeding on probing; CAL, clinical attachment loss; PPD, probing pocket depth, *p < 0.05.

These findings suggest that elevated PPAD enzymatic activity, strongly linked to specific clusters of polymorphic variants of the *ppad* gene, may contribute to destruction of periodontal tissue, as reflected by worsened clinical parameters of periodontium. This observation highlights potential role of PPAD, and particularly its specific genetic variants in bacterial virulence and modulation of host**-**pathogen interactions in the clinical course of PD.

### 3.7 *In vitro* studies

#### 3.7.1 Standardization of MC3T3-E1 cell model

**Figure 4:**
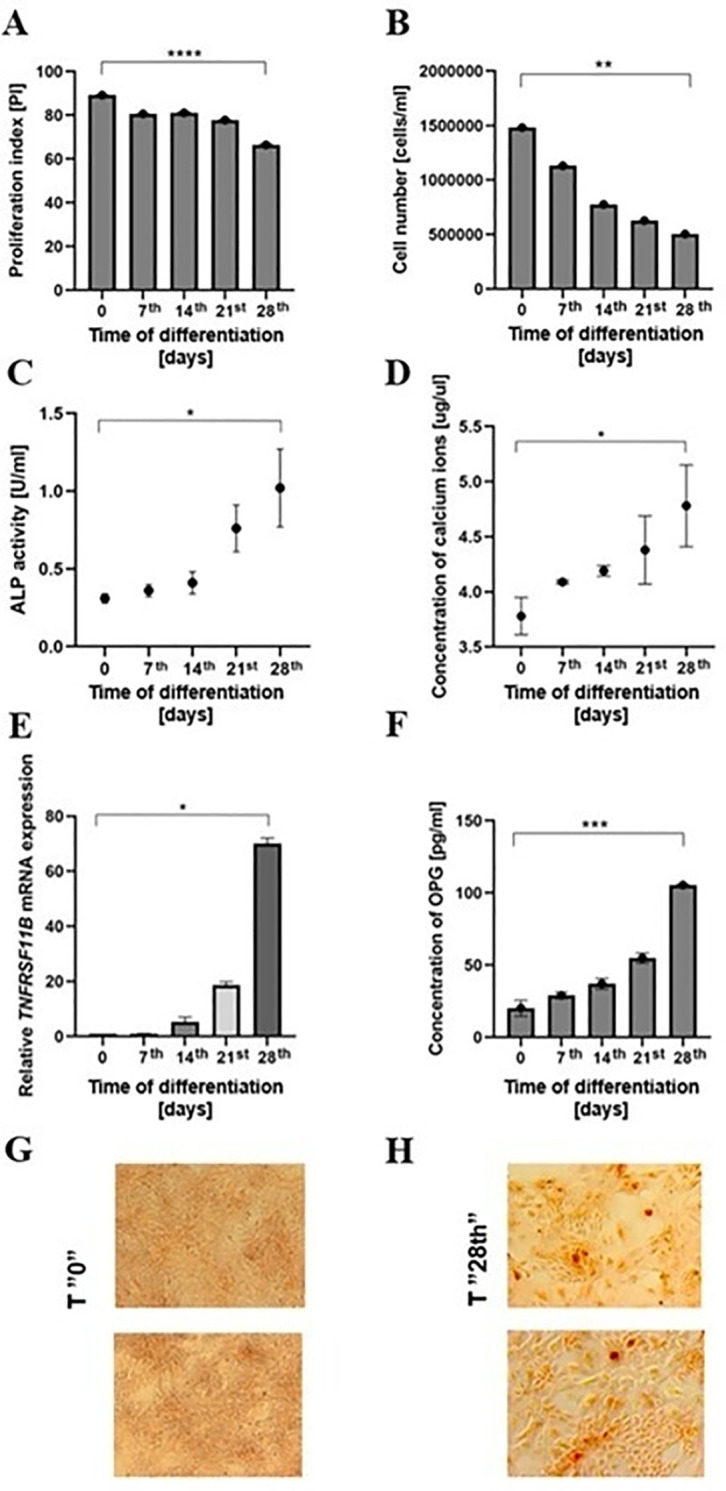
Differentiation of the murine MC3T3 pre-osteoblasts to osteoblasts. Murine MC3T3**-**E1 pre**-**osteoblasts were differentiated toward mature osteoblasts for 28 days by α**-**MEM supplementation with osteogenic factors (10^-8^ M dexamethasone, 2 mM β**-**glycerophosphate, and 20 μg/mL ascorbic acid) and cells cultured at day “0” w/o osteogenic factors used as a control. Cell proliferation was assessed using **(A)** a colorimetric MTT assay, and **(B)** manual cell counting; **(C)** Alkaline phosphatase (ALP) activity and **(D)** calcium ions concentration were measured in cell culture supernatants using a colorimetric enzymatic assay and a colorimetric calcium assay, respectively; **(E)** Relative mRNA expression of TNF Receptor Superfamily Member 11b (*TNFRSF11B)* was determined by qRT**-**PCR with *β-actin* used as a reference gene; **(F)** Osteoprotegerin (OPG) concentration was measured in cell culture supernatants using a colorimetric assay; Representative images of Alizarin red staining **(G)** at day “0” (control, no osteogenic factors added), and **(H)** at day 28^th^ for detection of mineralization nodules are presented. Results were obtained from three independent experiments. Data show mean values ± SD. Statistical analysis was performed using a paired Student’s *t***-**test. *p < 0.05; **p < 0.01; ***p < 0.001; ****p < 0.0001.

At first stage, murine pre-osteoblast cultures were treated over a 28**-**day period with osteogenic factors to acquire the phenotype of mature osteoblasts. All analyses were carried out at 7^th^, 14^th^, 21^st^, and 28^th^ day of culture and at day “0” (control, no osteogenic factors added). In line with the expected effects of dexamethasone, a gradual decline of both, total cell number and proliferation index was observed throughout the differentiation period of 28 days (**Figures 4A, B**). To assess further osteoblast maturation, enzymatic activity of ALP and concentration of Ca^2+^, two well**-**established markers of osteogenesis were measured. ALP activity increased progressively throughout the experiment, peaking at day 28^th^ (**Figure 4C**), and Ca^2+^ concentration showed a steady rise over the 28 days of differentiation, reaching the highest level at the final time point, further supporting mineralization process (**Figure 4D**). Gene expression analysis of TNF Receptor Superfamily Member 11b (*TNFRSF11B)* encoding OPG and protein quantification of OPG were performed to assess the regulatory mechanisms of bone resorption during differentiation. Both, the *TNFRSF11B* mRNA levels and secreted OPG concentrations increased over time with maximum expression observed at 28^th^ day (**Figures 4E, F**). Given the critical role of OPG as an inhibitor of osteoclastogenesis and mediator of bone homeostasis, these results reflect the full maturation of the osteoblast phenotype. Finally, calcium deposits indicative of mineralized matrix formation were visualized using Alizarin Red staining. We observed progressive formation and expansion of mineralization nodules over time with successful osteogenic differentiation after 28 days of experiment duration. Control cultures not exposed to differentiation factors (day “0”) showed no mineralization, validating the experimental setup (**Figures 4G, H**).

#### 3.7.2 Analysis of virulence of *P. gingivalis* strains producing 3 most frequently occurring clusters of polymorphic variants of *ppad* gene - studies in murine osteoblasts

To assess virulence of *P. gingivalis* strains which revealed elevated values of PPAD activity (**>** 200%), and produced any of 3 most frequently occurring clusters of polymorphic variants: i) S191F + N291D, ii) S191F + N291D + S528G, or iii) S203P + G231N, E232T, N235D + N291D + A515V + S528G (**Table 5**, **Figure 2B**), the analysis of *COX-2*, *IL-1β* and OPG in murine (MC3T3**-**E1) osteoblasts was performed.

After 24 h infection with relevant *P. gingivalis* strains, relative expression of *COX- 2* and *IL- 1β* was quantified and compared to the expression observed after infection with the reference *P. gingivalis* ATCC 33277 and the strains w/o polymorphic variants of *ppad*.

**Figure 5:**
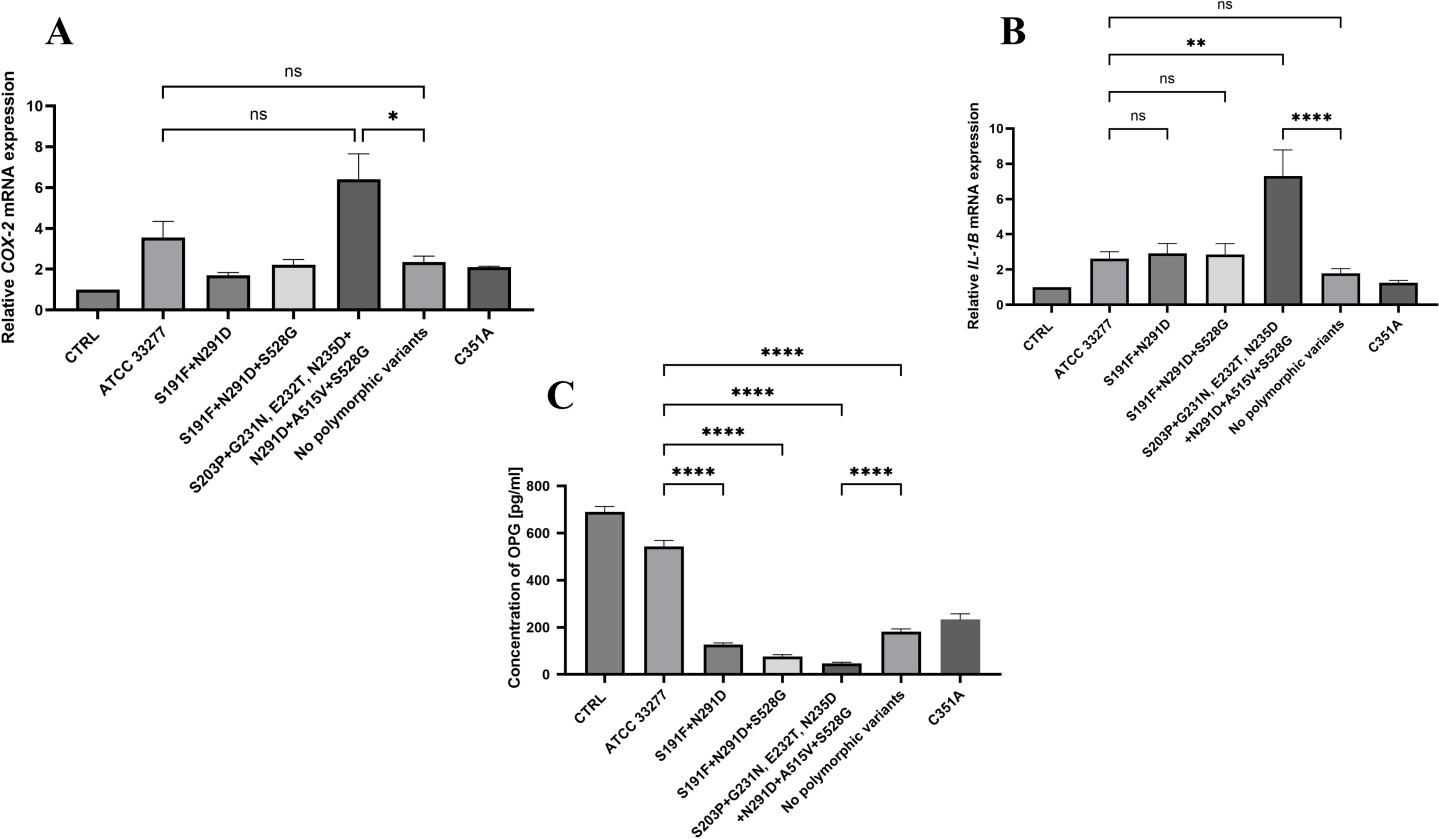
Analysis of pro-inflammatory and anti-resorptive factors in osteoblasts infected with clinical *P. gingivalis* strains carrying clusters of polymorphic variants of *ppad*. Relative expression level of **(A)** cyclooxygenase-2 (*COX-2*), **(B)** interleukin-1β (*IL-1β*), and **(C)** concentration of osteoprotegerin (OPG) in murine osteoblast (MC3T3-E1) cultures at 28^th^ day of differentiation, infected for 24 h at MOI of 100 with *P. gingivalis* strains carrying any of 3 most frequently occurring clusters of polymorphic variants of the *ppad* gene: i) S191F + N291D, ii) S191F + N291D + S528G, or iii) S203P + G231N, E232T, N235D + N291D + A515V + S528G. CTRL, control, uninfected MC3T3-E1 osteoblasts at 28^th^ day of differentiation; ATCC 33277, *P. gingivalis* reference strain; C351A, a control ATCC 33277 strain, which produces a catalytically inactive form of PPAD; qRT-PCR was performed using *β*-actin as a reference gene. Data represent the mean ± SEM from three independent experiments. Statistical analysis was performed using **(A)** the Kruskal-Wallis test with Dunn’s *post hoc* test or **(B, C)** one-way ANOVA with Tukey’s *post hoc* test. *p < 0.05; **p < 0.01; ****p < 0.0001; ns, not significant.

Meaningful upregulation of *COX- 2* (~6**-**fold) was observed in osteoblasts infected with *P. gingivalis* strains carrying the most complex cluster of polymorphic variants of *ppad* (S203P + G231N, E232T, N235D + N291D + A515V + S528G) and it was significantly elevated in comparison to expression induced by *P. gingivalis* strains w/o polymorphic variants of *ppad* (*p* < 0.05). Conversely, strains producing any of two remained clusters (S191F + N291D or S191F + N291D + S528G) induced only moderate (~1.5**-**fold and ~2**-**fold, respectively) upregulation of *COX-2* expression (**Figure 5A**). Furthermore, *P. gingivalis* strains carrying the cluster of S203P + G231N, E232T, N235D + N291D + A515V + S528G variants induced nearly 7**-**fold the expression of *IL- 1β* in comparison with its expression after infection with *P. gingivalis* ATCC 33277 (*p* < 0.01) and after infection with strains not harboring polymorphic variants of *ppad* gene (*p* < 0.0001). Expression of *IL- 1β* after osteoblasts infection with two other clusters of *ppad* variants (S191F + N291D or S191F + N291D + S528G) was comparable with expression observed after *P. gingivalis* ATCC 33277 infection and slightly elevated in comparison with expression observed after cells infection by *P. gingivalis* strains w/o polymorphic variants of *ppad* (**Figure 5B**).

Further, we analyzed the concentration of OPG, also known as osteoclastogenesis inhibitory factor. The most pronounced reduction of OPG concentration was observed after osteoblasts infection with *P. gingivalis* strains carrying the cluster of S203P + G231N, E232T, N235D + N291D + A515V + S528G variants (94% of control, uninfected cells), next by *P. gingivalis* strains carrying the cluster of S191F + N291D + S528G (89% of control), and finally by strains producing the cluster of S191F + N291D variants (82% of control). In case of all clusters, the decrease of OPG concentration was significant compared with its expression in cells infected with reference wild**-**type *P. gingivalis* ATCC 33277 strain (*p* < 0.0001). OPG concentration in cells treated with any of 3 clusters of polymorphic variants was also reduced in comparison with its expression after infection with *P. gingivalis* strains w/o polymorphic variants of *ppad*, however, significant reduction of OPG concentration was observed after cells treatment with strains containing the cluster of S203P + G231N, E232T, N235D + N291D + A515V + S528G variants (*p* < 0.0001) (**Figure 5C**). To exclude the possibility that observed effects could be attributed to cytotoxicity due to the infection, murine osteoblasts were infected for 24 h (MOI 100) with *P. gingivalis* strains expressing 3 most frequently occurring clusters of polymorphic variants: i) S191F + N291D, ii) S191F + N291D + S528G, or iii) S203P + G231N, E232T, N235D + N291D + A515V + S528G and cell viability was assessed using the ATCC 33277 as reference strain and uninfected osteoblasts as control. Infection with each tested *P. gingivalis* strain had negligible effects on cell viability as determined by MTT assay (**Supplementary Figure 1**, **Supplementary Data**). These data suggest that *P. gingivalis* strains expressing the cluster of S203P + G231N, E232T, N235D + N291D + A515V + S528G variants of *ppad*, exert pro**-**inflammatory effects on osteoblasts and may promote bone resorption via *RANKL*/*RANK*/*OPG* pathway modulation.

#### 3.7.3 Analysis of virulence of *P. gingivalis* strains carrying the cluster of S203P + G231N, E232T, N235D + N291D + A515V + S528G variants of *ppad* gene - studies in human gingival fibroblasts

Next, we continued evaluation of virulence of *P. gingivalis* strains with elevated levels of PPAD activity (**>** 200%), which produced one of most frequently occurring clusters of polymorphic variants of *ppad* (S203P + G231N, E232T, N235D + N291D + A515V + S528G) (**Table 5**, **Figure 2B**). To this aim two sets of experiments were carried out employing PHGFs from donor diagnosed with advanced and with moderate PD and tested the immune response of cells infected with relevant *P. gingivalis* strains by analysis of *TNF-α* and *IL-6* expression.

**Figure 6:**
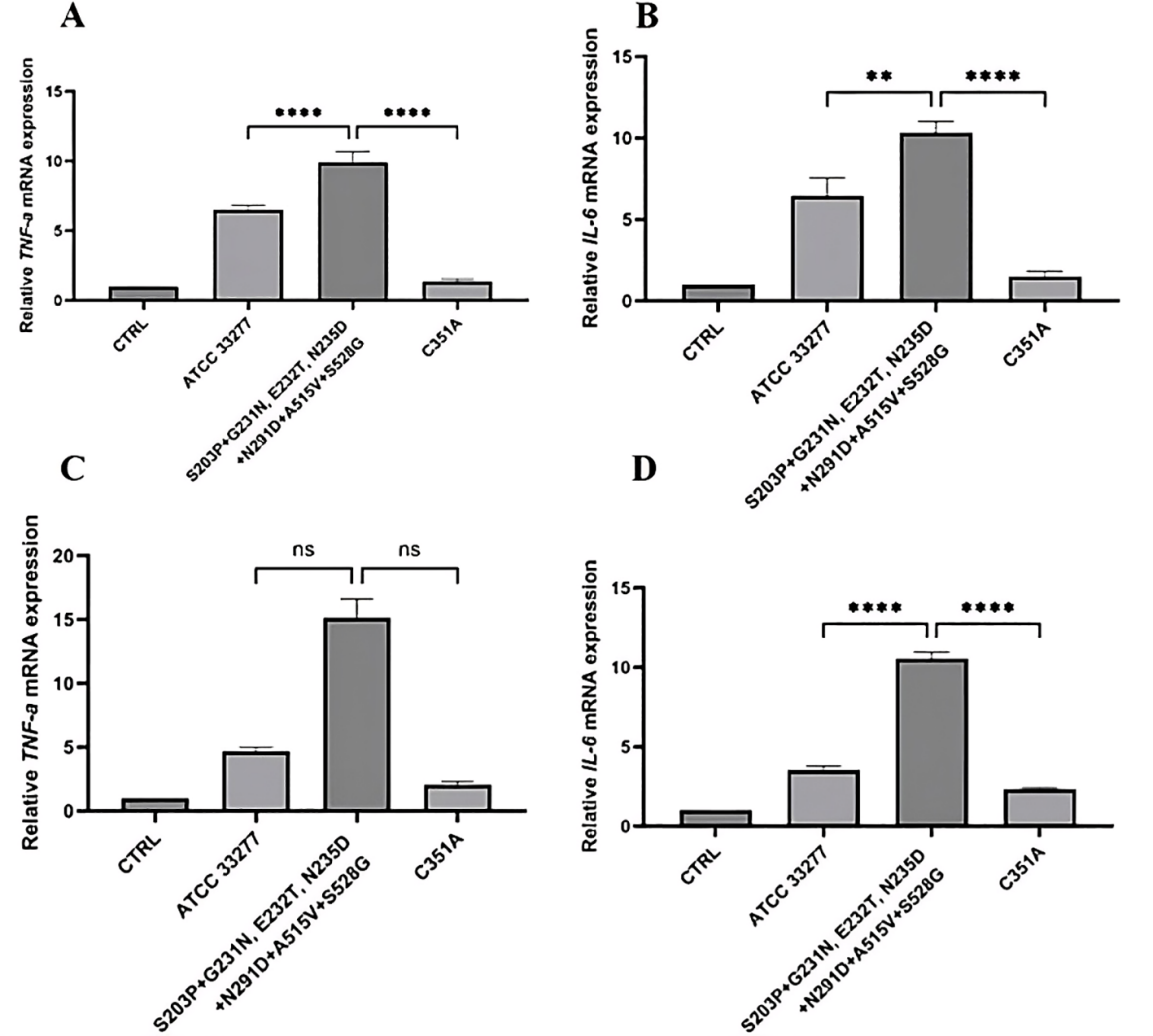
Analysis of pro-inflammatory cytokines expression by PHGFs infected with clinical *P. gingivalis* strains carrying one of most frequently occurring clusters of polymorphic variants of *ppad*. Relative expression levels of **(A, C)** tumor necrosis factor α (*TNF-α)*, and **(B, D)** interleukin-6 (*IL-6*) in PHGFs from **(A, B)** advanced PD or **(C, D)** moderate PD infected for 24 h at MOI of 100 with *P. gingivalis* strains carrying one of 3 most frequently occurring clusters of polymorphic variants of the *ppad* gene: iii) S203P + G231N, E232T, N235D + N291D + A515V + S528G. CTRL, control, uninfected primary human gingival fibroblasts (PHGFs); ATCC 33277, *P. gingivalis* reference strain; C351A, a control ATCC 33277 strain, which produces a catalytically inactive PPAD; qRT-PCR was performed using *β*-actin as a reference gene. Data represent the mean ± SEM from three independent experiments. Statistical analysis was performed using **(A, C)** Kruskal-Wallis test with Dunn’s *post hoc* test or **(B, D)** one-way ANOVA with Tukey’s *post hoc* test. **p < 0.01; ****p < 0.0001; ns, not significant.

Infection of PHGFs from donor with advanced PD by *P. gingivalis* strains harboring the cluster of S203P + G231N, E232T, N235D + N291D + A515V + S528G variants resulted in ~10**-**fold upregulation of *TNF-α* and *IL-6* expression in comparison to their expression in control cells; expression of both genes increased significantly in comparison to response of cells infected with *P. gingivalis* reference strain ATCC 33277 (*TNF-α p* < 0.0001; *IL-6 p* < 0.01) and after infection with control mutant strain producing inactive PPAD (*TNF-α p* < 0.0001; *IL-6 p* < 0.0001) (**Figures 6A, B**). Following infection of PHGFs from moderate PD, expression of both genes was upregulated compared to ATCC 33277 strain and C351A, but statistically significant only in case of *IL- 6* (*p* < 0.0001) (**Figures 6C, D**).

**Figure 7:**
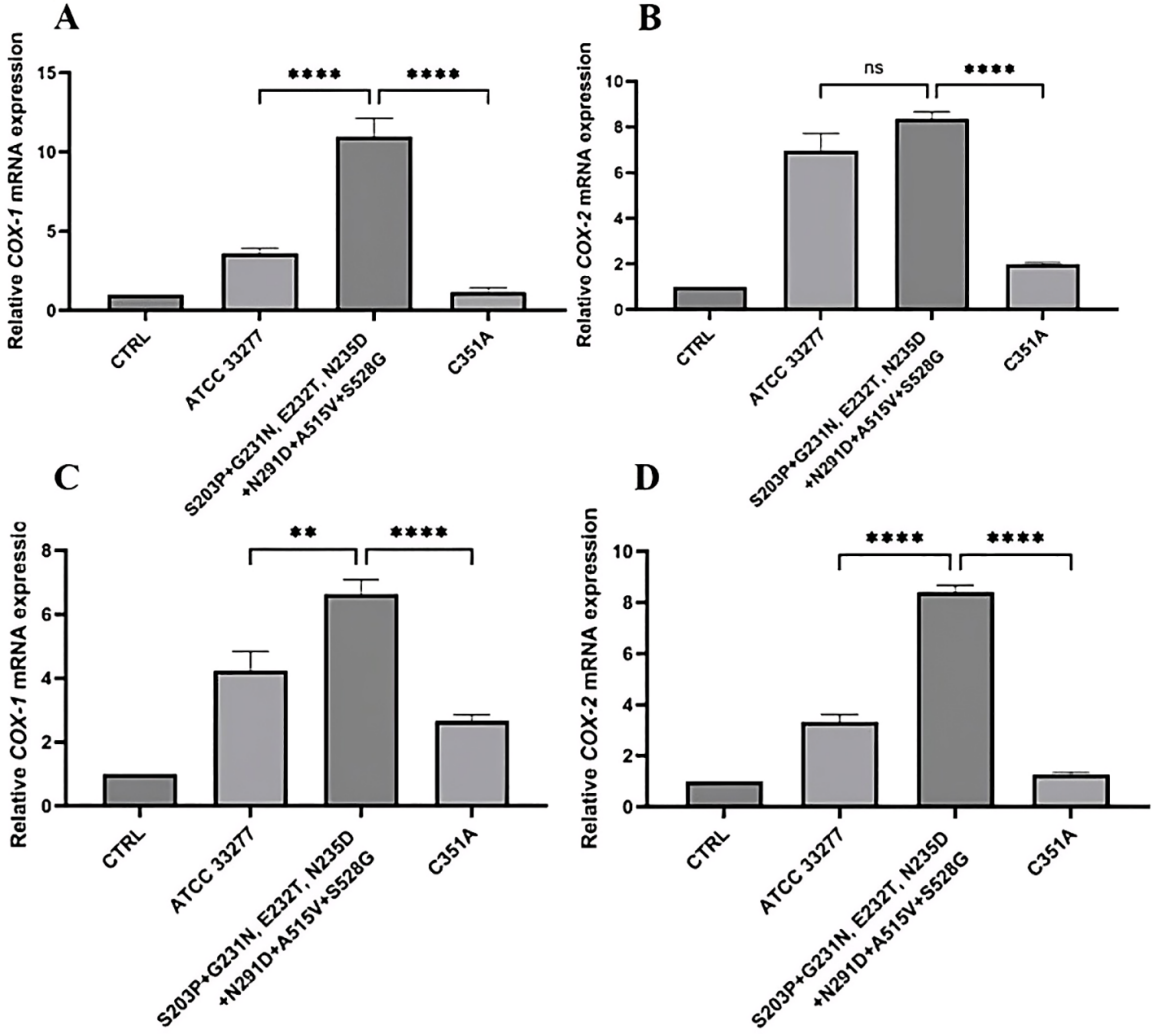
Analysis of cyclooxygenases expression by PHGFs infected with clinical *P. gingivalis* strains carrying one of most frequently occurring clusters of polymorphic variants of *ppad*. Relative expression levels of **(A, C)** cyclooxygenase-1 (*COX-1)*, and **(B, D)** cyclooxygenase-2 (*COX-2*) in PHGFs from **(A, B)** advanced PD or **(C, D)** moderate PD infected for 24 h at MOI of 100 with *P. gingivalis* strains carrying one of 3 most frequently occurring clusters of polymorphic variants of the *ppad* gene: iii) S203P + G231N, E232T, N235D + N291D + A515V + S528G. CTRL, control, uninfected primary human gingival fibroblasts (PHGFs); ATCC 33277, *P. gingivalis* reference strain; C351A, a control ATCC 33277 strain, which produces a catalytically inactive PPAD; qRT-PCR was performed using *β*-actin as a reference gene. Data represent the mean ± SEM from three independent experiments. Statistical analysis was performed using one-way ANOVA with Tukey’s *post hoc* test. **p < 0.01; ****p < 0.0001; ns, not significant.

Considering the crucial impact of the PGE_2_**-**dependent pathway in PD pathogenesis, we analyzed whether *P. gingivalis* strains producing the S203P + G231N, E232T, N235D + N291D + A515V + S528G variants may affect the expression of *COX-1* and *COX-2*. Cells infection by *P. gingivalis* strains producing this cluster of variants resulted in significant upregulation of *COX****-****1* as compared to infection with *P. gingivalis* ATCC 33277 (PHGFs, advanced PD *p* < 0.0001; PHGFs, moderate PD *p* < 0.01) and inactive C351A strain (PHGFs, advanced and moderate PD *p* < 0.0001) (**Figures 7A, C**). Similarly, *P. gingivalis* carrying most frequently occurring polymorphic variants of *ppad* also induced *COX- 2* expression by PHGFs from advanced and moderate PD (*p* < 0.0001, compared to inactive mutant C351A) and by PHGFs from moderate PD (*p* < 0.0001, compared to *P. gingivalis* ATCC 33277) (**Figures 7B, D**). In line with previous observations in murine osteoblasts, *P. gingivalis* strains, which produced one of most frequently occurring clusters of polymorphic variants of *ppad* (S203P + G231N, E232T, N235D + N291D + A515V + S528G) had no effect on PHGFs viability (**Supplementary Figure 1**, **Supplementary Data**).

Collectively, these findings demonstrated that clinical *P. gingivalis* strains carrying one of most frequently occurring clusters of polymorphic variants of *ppad* (S203P + G231N, E232T, N235D + N291D + A515V + S528G) modulate the expression of inflammatory genes of PHGFs, which may suggest a genetic variant**-**dependent effect on host immune response and pathogenicity of *P. gingivalis*.

## 4 Discussion

PD is a chronic inflammatory disease affecting over 50% of the global adult population, with a multifactorial etiology, including both, microbial and host-related factors (Geraldino-Pardilla et al., 2017; Olsen et al., 2018; Tonetti et al., 2017). Among the key bacterial contributors, *P. gingivalis*, which is a member of the “red complex” bacteria, has been extensively studied due to its wide array of virulence factors and its capacity to modulate host immune response (Aliko et al., 2018; Ferenc et al., 2026; Gawron et al., 2014; Gully et al., 2014). In recent years, considerable attention has been concentrated on PPAD, particularly due to its unique ability to catalyze citrullination, a post**-**translational modification of bacterial and/or host proteins which may exert deleterious effects on host immunity and periodontal tissue healing (Bereta et al., 2024; Gawron et al., 2014, 2017; Pyrc et al., 2013). Moreover, genetic heterogeneity of virulence**-**associated genes produced by *P. gingivalis* is increasingly recognized as a contributing factor of adaptability and variable impact of this pathogen on clinical course of PD. In previous work we identified and described a super**-**active G231N, E232T, N235D variant of *ppad* produced by *P. gingivalis* strains and colonizing advanced PD suggesting it as a potential candidate for novel targets for supportive therapy of PD. Furthermore, a comprehensive analysis of *ppad* gene sequences expressed by *P. gingivalis* strains from PD vs. healthy individuals compared with public databases was reported, indicating its considerable heterogeneity among individual *P. gingivalis* strains, in particular from PD (Bereta et al., 2024; Strzelec et al., 2025).

In this study, we provide new evidence for the existence of 3 clusters of polymorphic variants of *ppad* gene produced by *P. gingivalis* strains which presented a meaningful increase of activity values of PPAD. Moreover, we demonstrated that occurrence of any of newly identified clusters correlated with increased values of clinical parameters of periodontium from PD, upregulated expression of pro**-**inflammatory cytokines and reduced concentration of osteoclastogenesis inhibitory factor.

Sequencing analysis of *ppad* gene expressed by *P. gingivalis* strains from PD showed multiple nucleotide substitutions classified as polymorphic variants, *e.g.*, S191F, S203P, G231N, E232T, N235D, N291D, A515V, and S528G, opposite to *P. gingivalis* strains from healthy donors; these observations are consistent with findings from our previous studies performed in other cohort of PD donors, and suggest the reproducibility of *ppad* sequence variability in disease**-**associated strains (Bereta et al., 2024; Strzelec et al., 2025). In contrast, in the study reported by Gabarrini et al. (2015), no substitutions resulting in amino acid changes in the PPAD protein were detected (Gabarrini et al., 2015). This discrepancy is likely attributable to methodological limitations since the authors analyzed only 7 of 92 available clinical strains and relied on restriction enzyme digestion, an indirect approach lacking sufficient resolution to detect sequence-level variability. On the contrary, our study employed full**-**length sequencing of the *ppad* gene from *P. gingivalis* strains isolated from both, PD patients and controls with healthy periodontium using the Sanger method, followed by *in silico* comparison with a reference wild**-**type strain of *P. gingivalis*. This comprehensive strategy enables accurate identification of nucleotide substitutions and polymorphic variants that may be overlooked by partial or indirect techniques.

Most notably, a 7**-**nucleotide substitution resulting in three amino acid changes (G231N, E232T, N235D) located in close proximity to the PPAD active center was observed in approximately 25% of *P. gingivalis* strains from individuals with advanced PD. Strains carrying this *ppad* variant exhibited increased enzymatic activity, potentially leading to intensified local citrullination and enhanced release of inflammatory mediators (Bereta et al., 2024; Strzelec et al., 2025). Structural insights based on crystallographic models of PPAD indicate that these substitutions induce conformational changes within a loop adjacent to the active center. In particular, the E232T substitution affects a region essential for the deimination reaction, forming a channel through which water molecules enter and ammonia is released during citrullination. Replacement of the negatively charged glutamic acid with neutral threonine alters local charge and polarity, potentially influencing substrate access and product release. A similar effect on channel conformation may also exert the substitution of glycine to asparagine (G231N), which introduces a bulkier side chain in this region. Furthermore, the asparagine residue at position 235 is located within the channel responsible for hydroxyl ion passage, required to restore the active center to a catalytically competent state. Collectively, these amino acid substitutions are likely to modify the biochemical properties of PPAD leading to changes of its physiological function (Bereta et al., 2019; Montgomery et al., 2016).

The G231N, E232T, N235D substitutions have been associated with increased expression of pro**-**inflammatory mediators and more pronounced periodontal tissue destruction, supporting its direct contribution to bacterial virulence. Directed mutagenesis experiments further demonstrated that all three amino acid substitutions must be introduced simultaneously to generate a “super**-**active” variant of PPAD, highlighting the functional interdependence of these changes (Bereta et al., 2024). Structural and kinetic analyses have shown that such polymorphic combinations enhance catalytic turnover while altering substrate binding characteristics, reinforcing the concept that specific constellations of substitutions, rather than isolated single**-**site mutations, determine PPAD catalytic performance (Bereta et al., 2019). Our data are fully consistent with these observations and extend them by linking identified clusters of polymorphic variants with quantitative differences in both, *ppad* expression and PPAD enzymatic activity in clinical isolates of *P. gingivalis*.

Beyond mechanistic insights into PPAD function, these observations have also important methodological and clinical implications. The functional relevance of these observations is further supported by previous studies demonstrating that clinically significant PPAD variants can only be reliably identified through full**-**length *ppad* sequencing (Bereta et al., 2019). More recently, Kamińska et al. (2025) reported an association of the super**-**active PPAD variant and distinct oral microbiota profiles in PD patients, indicating that the variability of *ppad* sequence may influence disease pathophysiology beyond enzymatic activity alone (Kaminska et al., 2025). Taken together, these findings suggest that studies based on partial or indirect methodologies likely underestimated the true diversity and functional relevance of PPAD variants.

Another polymorphic variant identified in over half (51.43%) of the analyzed PD derived *P. gingivalis* strains was the N291D substitution. It is located in close proximity to the PPAD active center, near residue Asn297, suggesting a potential functional relevance (Strzelec et al., 2025). Replacement of a neutral asparagine with a negatively charged aspartic acid may alter the local electrostatic environment of the catalytic site, thereby affecting substrate binding or catalytic efficiency. Such a charge redistribution could influence hydrogen bonding networks or introduce new electrostatic interactions, potentially stabilizing or destabilizing the active site conformation (Betts and Russell, 2003; DePristo et al., 2005). Although these effects remain hypothetical, they are supported by structural considerations and warrant further validation using crystallographic or molecular dynamics approaches.

Other polymorphic variants identified as a single substitution or in clusters were the S191F and S528G substitutions. The S191F substitution introduces hydrophobic phenylalanine residue in place of serine, which may influence local protein folding or stability. In turn, the S528G variant removes a polar side chain and increases local flexibility of the protein backbone, which could affect long**-**range conformational dynamics of PPAD (Betts and Russell, 2003; DePristo et al., 2005). Although these variants are located more distally from the PPAD active center compared to the G231N, E232T, N235D, and N291D variants, their recurrent co**-**occurrence with mutations proximal to the active site may indicate a possible cooperative effect on enzyme structure and function.

At the expression and functional levels, clinical *P. gingivalis* strains derived from PD patients displayed marked heterogeneity in relative *ppad* expression, with more than half of the analyzed strains exhibiting 2**-**fold higher expression levels compared with the reference ATCC 33277 strain. Notably, the majority of these high**-**expressing strains harbored polymorphic variants of the *ppad* gene, whereas strains carrying only missense or synonymous substitutions showed variable expression without a consistent trend toward up**-** or down**-**regulation. A parallel pattern was observed with respect to PPAD enzymatic activity. Importantly, further analyses showed that elevated activity of PPAD was associated with three clusters of polymorphic variants, including i) S191F + N291D; ii) S191F + N291D + S528G; and iii) S203P + G231N, E232T, N235D + N291D + A515V + S528G. These observations indicate that PPAD activity is modulated by combination of specific substitutions, supporting a cumulative and/or synergistic effect on enzyme function. This interpretation is consistent with earlier structural and functional studies demonstrating that specific PPAD variants, particularly substitutions in proximity to the catalytic center, exhibit increased enzymatic activity (Bereta et al., 2019). Although data on PPAD polymorphic variants remain limited, studies on other bacterial enzymes demonstrate that the functional consequences of mutations often depend on their specific combinations rather than on individual substitutions. For example, synergistic and epistatic interactions between distinct amino acid changes in TEM**-**1 and CTX**-**M β**-**lactamases have been shown to markedly alter enzymatic activity and substrate specificity in a manner not predictable from single mutations alone (Dellus-Gur et al., 2015; Patel et al., 2018). In this context, our findings suggest that a similar cumulative effect of clusters of specific substitutions also operates in *P. gingivalis* PPAD, underscoring the importance of considering variant constellations when evaluating the functional and pathogenic relevance of PPAD diversity.

Furthermore, we examined whether increased PPAD activity of *P. gingivalis* strains carrying any of the three clusters of polymorphic variants, *i.e.*, i) S191F + N291D; ii) S191F + N291D + S528G; and iii) S203P + G231N, E232T, N235D + N291D + A515V + S528G is associated with clinical severity of PD. We found that *P. gingivalis* strains exhibiting the highest PPAD activity predominantly derived from PD patients presenting with more severe inflammation and destruction of periodontium. Although these findings represent one of the first clinical genotype**-**phenotype associations, they are supported by previous molecular studies linking identification of the G231N, E232T, N235D variant with worse condition of periodontium of patients diagnosed with advanced PD as well as by experimental animal models demonstrating reduced inflammation and bone loss following infection with PPAD**-**knockout *P. gingivalis* strains (Bereta et al., 2024; Gully et al., 2014). At the same time, clinical heterogeneity remains evident, as recent reports indicate that the impact of super**-**active PPAD variants on periodontal parameters may depend on host**-**related factors, microbial context, or cohort size (Kaminska et al., 2025). Broader analyses of *P. gingivalis* virulence further highlight that PPAD acts synergistically with other virulence determinants, including gingipains and host immune pathways, to promote chronic inflammation and tissue degradation, mechanisms that may amplify clinical severity in the presence of highly**-**active PPAD variants (Chow et al., 2022).

Finally, *in vitro* host cell**-**bacterial assays provided direct experimental evidence linking elevated PPAD activity of *P. gingivalis* strains carrying clusters of specific co**-**occurring polymorphic variants to enhanced host inflammatory and bone**-**related responses. The downstream inflammatory response was analyzed by measuring *COX-2* and *IL-1β* expression, which constitute well**-**established indicators of pro**-**inflammatory signaling and mediators of tissue destruction in PD (Gawron et al., 2014; Strzelec et al., 2025). COX-2 catalyzes PGE_2_ production, which promotes vasodilation, leukocyte recruitment and osteoclast differentiation, while IL-1β stimulates inflammatory cascades and contributes to bone resorption (Båge et al., 2011). Infection of osteoblasts with *P. gingivalis* strains carrying clusters of specific *ppad* variants induced markedly expression of *COX****-****2* and *IL-1β* and reduced osteoprotegerin levels, indicating a shift toward increased osteoclastogenesis and bone resorption. These observations are consistent with previous reports, which showed that *P. gingivalis* infection can upregulate RANKL expression and reduce osteoprotegerin in osteoblasts, thereby promoting osteoclast activation and bone resorption (Le et al., 2009; Okahashi et al., 2004). Similarly, gingival fibroblasts from PD donors exposed to the same high**-**activity PPAD variant strains exhibited pronounced upregulation of pro**-**inflammatory mediators, *i.e.*, *TNF-α*, *IL-6*, *COX-1*, and *COX-2*, relative to the reference and variant**-**free strains of *P. gingivalis* and aligns with literature indicating that PPAD activity can amplify TLR2**-**dependent inflammatory signaling and contribute to chronic periodontal inflammation (Wielento et al., 2022). The pathogenic significance of PPAD variability is further underscored by the unique biochemical properties of this enzyme. PPAD is the only known bacterial peptidylarginine deiminase capable of targeting C**-**terminal arginine residues, leading to the generation of citrullinated peptides with potential implications for host**-**pathogen interactions, chronic inflammation in PD, and possibly RA through neoantigen formation and immune modulation (Gabarrini et al., 2015; Montgomery et al., 2016). The *in vitro* findings presented in this study provide important insight into the functional consequences of *ppad* gene variability in the context of host**-**pathogen interactions. The observed differences in *COX-2* and *IL-1β* expression among clinical strains indicate that not all *P. gingivalis* isolates exert the same pro-inflammatory potential, supporting the concept of strain**-**specific virulence. A possible explanation of such heterogeneity lies in differences of PPAD activity and its interaction with other virulence factors, such as for example fimbriae. *P. gingivalis* is characterized by substantial genomic diversity, including variation in virulence**-**associated genes, mobile genetic elements, and surface structures, such as fimbrial adhesions (Watanabe et al., 2011). The *fimA* gene, encoding major long fimbriae, exhibits allelic variation that defines at least six genotypes (I**-**V, Ib) with documented differences in adhesion and invasion capacities, indicating that surface structure diversity contributes to strain**-**specific pathogenic properties (Enersen et al., 2013). It has been demonstrated that PPAD enzymatic activity, together with fimbrial structures, is required for efficient activation of TLR2**-**dependent signaling pathways (Wielento et al., 2022). This suggests that citrullination mediated by PPAD may modify bacterial or alternatively host**-**derived proteins, enhancing their recognition by innate immune receptors and amplifying inflammatory responses (Wielento et al., 2026). In this context, sequence variation in the *ppad* gene identified in this study may translate into functional divergence in the ability of individual strains to activate downstream inflammatory pathways. This provides a mechanistic link between genetic diversity within *P. gingivalis* populations and heterogeneity in host immune responses observed in PD (Wielento et al., 2022, 2026). Furthermore, the differential induction of inflammatory markers in PHGFs highlights the importance of considering strain**-**level variability when investigating pathogen-driven inflammation. These findings support the hypothesis that specific *ppad* variants may be associated with differences in inflammatory potential and host immune activation, which may correspond with PD progression (Wielento et al., 2023). Therefore, the observed upregulation of *COX-2* and *IL-1β* provides functional evidence that *ppad* gene variability can influence fibroblast**-**driven inflammatory pathways, linking genetic differences among strains to potential variations in host tissue damage.

Limitations of this study include analyses of samples collected from one clinical center, introducing potential selection bias. Additionally, differences in age and gender between study and control group donors may represent confounding factors. In the study group mostly individuals below 75 years of age were enrolled, with only two participants above this threshold. Although microbiome composition remains relatively stable in adults under 75, age**-**related effects cannot be completely excluded (LaMonte et al., 2019). Also, the PHGFs for *in vitro* experiments derived only from two PD patients and one control individual, which may limit generalizability despite technical replicates. Finally, the *in vitro* model does not fully reflect the complexity of the periodontal environment, including host**-**microbiome interactions and immune system contributions.

Despite these limitations, identification of functionally distinct PPAD variants indicates that genetic diversity within clinical strains of *P. gingivalis* could contribute to variability in PD progression and possibly treatment response. Further characterization of PPAD variants may facilitate the development of targeted diagnostic tools or therapeutic strategies aimed at host inflammatory responses modulation.

## 5 Conclusions

Collectively, our findings demonstrate that the presence of clusters of specific polymorphic variants of *ppad* gene drives elevated PPAD expression and enhanced enzymatic activity in clinical *P. gingivalis* strains. These genotype**-**dependent functional changes provide a mechanistic link between *ppad* sequence variation, PPAD catalytic properties, and amplified host inflammatory and bone**-**related responses. Consequently, specific combinations of PPAD polymorphic variants are likely to directly contribute to periodontal tissue destruction, which is supported by previous biochemical and *in vivo* reports of PPAD**-**mediated pathogenicity.

## Data Availability

The datasets presented in this study can be found in online repositories. The names of the repository/repositories and accession number(s) can be found below: https://www.ncbi.nlm.nih.gov/, PQ605366 PQ605367 PQ605368 PQ605369 PQ605370 PQ605371 PQ605372 PQ605373 PQ605374 PQ605375 PQ605376 PQ605377 PQ605378 PQ605379 PQ605380 PQ605381 PQ605382 PQ605383 PQ605384 PQ605385 PQ605386 PQ605387 PQ605388 PQ605389 PQ605390 PQ605391 PQ605392 PQ605393 PQ605394 PQ605395 PQ605396 PQ605397 PQ605398 PQ605399 PQ605400 PP079941 PP079942 PP079943 PP079944 PP079945 PP079946 PP079947 PP079948 PP691522 PP691523 PP691524 PP691525 PP691526 PP691527 PP691528 PQ605401 PQ605402 PQ605403 PQ605404 PQ605405.

## Supplementary material

Supplementary Figure 1 Cell viability. Viability determined by MTT assay of **(A)** murine osteoblasts at 28^th^ day of differentiation, infected for 24 h at MOI of 100 with *P. gingivalis* strains carrying either of 3 clusters of polymorphic variants of the *ppad* gene: i) S191F + N291D, ii) S191F + N291D + S528G, or iii) S203P + G231N, E232T, N235D + N291D + A515V + S528G, **(B)** PHGFs from advanced PD (n=1), and **(C)** PHGFs from moderate PD (n=1) infected for 24 h at MOI of 100 with *P. gingivalis* strains carrying one of 3 most frequently occurring clusters of polymorphic variants of the *ppad* gene: iii) S203P + G231N, E232T, N235D + N291D + A515V + S528G. CTRL, control, uninfected MC3T3-E1 osteoblasts at 28^th^ day of differentiation **(A)**; CTRL, control, uninfected primary human gingival fibroblasts (PHGFs) from advanced PD **(B)** and from moderate PD **(C)**; ATCC 33277, *P. gingivalis* reference strain; C351A, a control ATCC 33277 strain, which produces a catalytically inactive form of PPAD. Values representing changes in MTT processing are shown as mean ± SEM optical density at 560 nm. Assay was performed in three replicates.

Supplementary Table 1 Primer sequences used in the study (synthesized by Genomed S.A., Warsaw, Poland).

Supplementary Table 2 Accession numbers of GenBank sequences.
