# Supplementary material for: Pathogenicity of P. gingivalis strains carrying clusters of specific polymorphic variants of peptidylarginine deiminase gene
Source: Front Cell Infect Microbiol. 2026 May 12;16:1820133. doi: 10.3389/fcimb.2026.1820133 (PMC13201384; doi:10.3389/fcimb.2026.1820133)
Supplement: Supplementary Figure 1 — Cell viability. Viability determined by MTT assay of (A) murine osteoblasts at 28th day of differentiation, infected for 24 h at MOI of 100 with P. gingivalis strains carrying either of 3 clusters of polymorphic variants of the ppad gene: i) S191F + N291D, ii) S191F + N291D + S528G, or iii) S203P + G231N, E232T, N235D + N291D + A515V + S528G, (B) PHGFs from advanced PD (n=1), and (C) PHGFs from moderate PD (n=1) infected for 24 h at MOI of 100 with P. gingivalis strains carrying one of 3 most frequently occurring clusters of polymorphic variants of the ppad gene: iii) S203P + G231N, E232T, N235D + N291D + A515V + S528G. CTRL, control, uninfected MC3T3-E1 osteoblasts at 28th day of differentiation (A); CTRL, control, uninfected primary human gingival fibroblasts (PHGFs) from advanced PD (B) and from moderate PD (C); ATCC 33277, P. gingivalis reference strain; C351A, a control ATCC 33277 strain, which produces a catalytically inactive form of PPAD. Values representing changes in MTT processing are shown as mean ± SEM optical density at 560 nm. Assay was performed in three replicates. [file DataSheet1.doc]

# SUPPLEMENTARY DATA

**Supplementary Figures**

**
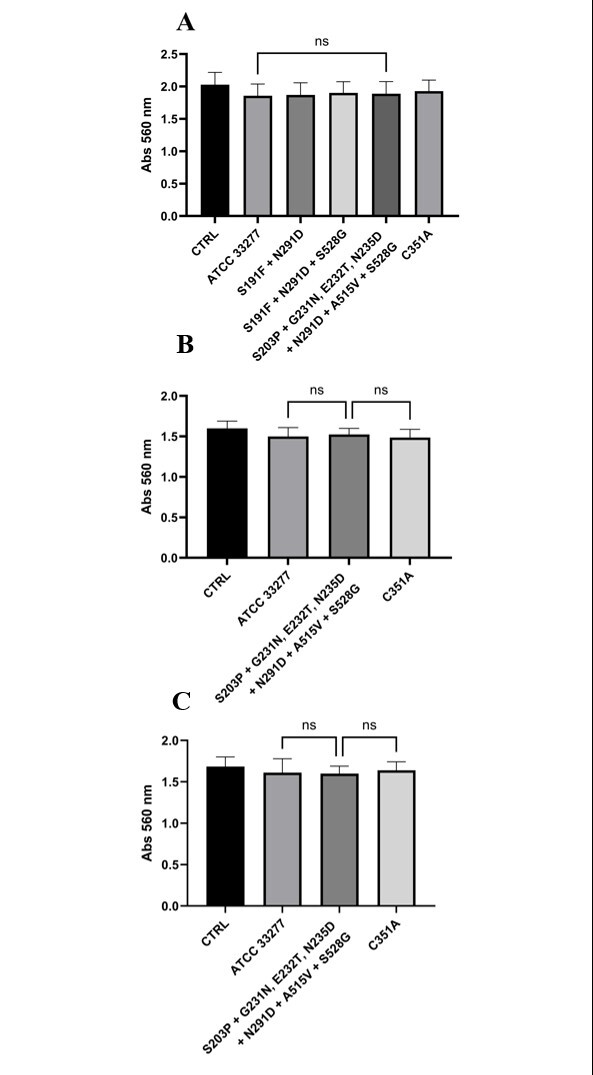
**

**Figure S1. Cell viability.** Viability determined by MTT assay of **(A)** murine osteoblasts at 28^th^ day of differentiation, infected for 24 h at MOI of 100 with *P. gingivalis* strains carrying either of 3 clusters of polymorphic variants of the *ppad* gene: i) S191F + N291D, ii) S191F + N291D + S528G, or iii) S203P + G231N, E232T, N235D + N291D + A515V + S528G, **(B)** PHGFs from advanced PD (n=1), and **(C)** PHGFs from moderate PD (n=1) infected for 24 h at MOI of 100 with *P. gingivalis* strains carrying one of 3 most frequently occurring clusters of polymorphic variants of the *ppad* gene: iii) S203P + G231N, E232T, N235D + N291D + A515V + S528G. CTRL, control, uninfected MC3T3-E1 osteoblasts at 28^th^ day of differentitation **(A)**; CTRL, control, uninfected primary human gingival fibroblasts (PHGFs) from advanced PD **(B)** and from moderate PD **(C)**; ATCC 33277, *P. gingivalis* reference strain; C351A, a control ATCC 33277 strain, which produces a catalytically inactive form of PPAD. Values representing changes in MTT processing are shown as mean ± SEM optical density at 560 nm. Assay was performed in three replicates.

**Supplementary Tables**

**Table S1:** Primer sequences used in the study (synthesized by Genomed S.A., Warsaw, Poland).

| **Primer name** | **Sequence 5’→3’** |
| --- | --- |
| *P. gingivalis* *16S rRNA*  (Bereta et al., 2024) | FOR: AGGCAGCTTGCCATACTGCG |
|  | REV: ACTGTTAGYAACTACCGATGT |
| *P. gingivalis* *ppad*_verification (full *ppad*)  (Bereta et al., 2024) | FOR: ATGAAAAAGCTTTTACAGGCTAAAGCCTTG |
|  | REV:TTATTTGAGAATTTTCATTGTCTCACGGATTCC |
| *P. gingivalis ppad_sequencing*  (This study) | FOR: TTGGCACCGAACAAAATCCT |
|  | REV: GTGTACGGTTGTTCATTGGT |
| *P. gingivalis 16S rRNA*  (qRT**-**PCR)  (Bereta et al., 2024) | FOR: AGGCAGCTTGCCATACTGCG |
|  | REV: ACTGTTAGYAACTACCGATGT |
| *P. gingivalis ppad*  (qRT**-**PCR)  (This study) | FOR: TGTACGATACGAACAAAGTAGGTC |
|  | REV: AATACTTGCCCCAACAGTCCAC |
| *TNF***-***α*  *(Homo sapiens)*  (This study) | FOR: CCCGAGTGACAAGCCTGTAG |
|  | REV: GATGGCAGAGAGGAGGTTGAC |
| *IL***-***6*  *(Homo sapiens)*  (This study) | FOR: ACAGCCACTCACCTCTTCAG |
|  | REV: CCATCTTTTTCAGCCATCTTT |
| *COX***-***1*  *(Homo sapiens)*  (This study) | FOR: CAGTTGCCAGATGCCCAGCTC |
|  | REV: GTGCATCAACACAGGCGCCTC |
| *COX***-***2*  *(Homo sapiens)*  (Gawron et al., 2014) | FOR: AGCCCTTCCTCCTGTGCCT |
|  | REV: GATGGCAGAGAGGAGGTTGAC |
| *TNFRSF11B  (Mus musculus)*  (This study) | FOR: GCCACGCAAAAGTGTGGAAT |
|  | REV: TTTGGTCCCAGGCAAACTGT |
| *IL***-***1β*  *(Mus musculus)*  (This study) | FOR: TGCCACCTTTTGACAGTGATG |
|  | REV: AAGGTCCACGGGAAAGACAC |
| *COX***-***2*  *(Mus musculus)*  (This study) | FOR: CAGCCAGGCAGCAAATCCTT |
|  | REV: GGGTGGGCTTCAGCAGTAAT |
| *β***-***actin*  *huACTB/moACTB*  *(Homo sapiens/Mus musculus)*  (Gawron et al., 2014) | FOR: CCACACTGTGCCCATCTACG |
|  | REV: AGGATCTTCATGAGGTAGTCAGTCAG |

**Table S2.** Accession numbers of GenBank sequences.

| Accession numbers of GenBank sequences analyzed in this study | |
| --- | --- |
| Strain name | Accession number |
| PD1 | PQ605366 |
| PD2 | PQ605367 |
| PD3 | PQ605368 |
| PD4 | PQ605369 |
| PD5 | PQ605370 |
| PD6 | PQ605371 |
| PD7 | PQ605372 |
| PD8 | PQ605373 |
| PD9 | PQ605374 |
| PD10 | PQ605375 |
| PD11 | PQ605376 |
| PD12 | PQ605377 |
| PD13 | PQ605378 |
| PD14 | PQ605379 |
| PD15 | PQ605380 |
| PD16 | PQ605381 |
| PD17 | PQ605382 |
| PD18 | PQ605383 |
| PD19 | PQ605384 |
| PD20 | PQ605385 |
| PD21 | PQ605386 |
| PD22 | PQ605387 |
| PD23 | PQ605388 |
| PD24 | PQ605389 |
| PD25 | PQ605390 |
| PD26 | PQ605391 |
| PD27 | PQ605392 |
| PD28 | PQ605393 |
| PD29 | PQ605394 |
| PD30 | PQ605395 |
| PD31 | PQ605396 |
| PD32 | PQ605397 |
| PD33 | PQ605398 |
| PD34 | PQ605399 |
| PD35 | PQ605400 |
| CTRL1 | PP079941 |
| CTRL2 | PP079942 |
| CTRL3 | PP079943 |
| CTRL4 | PP079944 |
| CTRL5 | PP079945 |
| CTRL6 | PP079946 |
| CTRL7 | PP079947 |
| CTRL8 | PP079948 |
| CTRL9 | PP691522 |
| CTRL10 | PP691523 |
| CTRL11 | PP691524 |
| CTRL12 | PP691525 |
| CTRL13 | PP691526 |
| CTRL14 | PP691527 |
| CTRL15 | PP691528 |
| CTRL16 | PQ605401 |
| CTRL17 | PQ605402 |
| CTRL18 | PQ605403 |
| CTRL19 | PQ605404 |
| CTRL20 | PQ605405 |
